# ASF1B promotes gastric cancer liver metastasis through inhibiting ZDHHC9/PCBP1/ SLC7A11 signaling axis mediated ferroptosis

**DOI:** 10.1038/s41698-026-01272-w

**Published:** 2026-01-14

**Authors:** Mingliang Wang, Kexun Yu, Mengdi Ma, Jing Li, Ying Zhang, Zhangyan Ke, Huizhen Wang, Yongxiang Li

**Affiliations:** 1 General Surgery Department, The First Affiliated Hospital of Anhui Medical University, Hefei, China; 2 Department of Pathology, The First Affiliated Hospital of Anhui Medical University, Hefei, China

**Keywords:** Cancer, Molecular biology, Biomarkers, Molecular medicine, Pathogenesis

## Abstract

Identifying potential molecular targets for GC liver metastasis (GCLM) may provide new treatment avenues. Initially, using label-free proteomics to screen clinical samples from GCLM patients suggested ASF1B as a possible promoter of GCLM. We further validated this finding with in vitro experiments and spleen injection liver metastasis model, subsequent transcriptome sequencing after ASF1B knockdown revealed SLC7A11-mediated ferroptosis is critical for GCLM progression. Mechanistically, ASF1B recruits and binds to the transcription factor HOXB3, thereby promoting ZDHHC9’s transcriptional level. Additionally, ZDHHC9 regulates SLC7A11-mediated ferroptosis in GC cells. Further tumor metastasis assays showed ZDHHC9 promotes peritoneal, pulmonary, and hepatic metastases in GC. Subsequently, immunoprecipitation and LC-MS analyses revealed the molecular interaction between ZDHHC9 and PCBP1. ZDHHC9, a palmitoyltransferase, inhibits ferroptosis by palmitoylating PCBP1. Mechanistically, ZDHHC9 palmitoylates PCBP1 at residue C109, inhibiting PCBP1 ubiquitination and thereby suppressing SLC7A11-mediated ferroptosis. In line with this, further experiments showed PCBP1 regulates ferroptosis by modulating SLC7A11 RNA stability. Finally, IHC and immunofluorescence revealed significant clinical correlations among ASF1B, ZDHHC9, PCBP1, and SLC7A11. Additionally, this signaling axis is strongly associated with PD-L1 expression. In conclusion, this study demonstrates ASF1B promotes GC liver metastasis by inhibiting ferroptosis via the ZDHHC9/PCBP1/SLC7A11 axis, providing a potential immunotherapeutic target for GCLM.

## Introduction

Gastric cancer (GC) is a highly aggressive malignancy of the digestive system, characterized by an insidious onset, rapid progression, and a propensity for metastasis, with the liver being the most common metastatic site^1,2^. Large-scale clinical studies indicate that while the 5-year survival rate for advanced GC undergoing radical surgery can exceed 70%, this rate drops below 30% once liver metastasis occurs, even with R0 resection^3,4^. Furthermore, patients with GCLM frequently develop resistance to chemotherapy^5^. Despite ongoing advancements in comprehensive treatment approaches, including combined immunotherapy and targeted therapies, the therapeutic outcomes for GCLM remain unsatisfactory^6^. Therefore, it is imperative to further investigate the intricate genetic and epigenetic mechanisms underlying GCLM to identify reliable molecular targets for therapy.

Post-translational modifications of proteins, such as palmitoylation, ubiquitination, SUMOylation, methylation, and phosphorylation, play pivotal roles in regulating a wide range of biological processes and may serve as key molecular mechanisms driving GCLM^7–10^. Among these, protein palmitoylation is a reversible lipid modification involving the covalent attachment of palmitic acid to the cysteine thiol groups of proteins, forming labile thioester bonds mediated by palmitoyl S-acyltransferases (PATs)^11,12^. This modification significantly enhances the hydrophobicity of specific protein subdomains, which can, in turn, influence protein localization and modulating various signaling pathways^13,14^. The ZDHHC family of proteins, comprising 23 members in mammals, with several members implicated in tumor progression. For instance, ZDHHC1 inhibits tumor growth by regulating cellular metabolism and oxidative stress^15^, while ZDHHC7 suppresses the malignant progression of prostate cancer cells by inhibiting androgen receptors^16^. The interplay between protein palmitoylation and ubiquitination was found in the present study. Ubiquitination, another critical post-translational modification which regulates specific proteins for degradation, thereby maintaining cellular homeostasis^17^. Similar to palmitoylation, ubiquitination is also reversible and dynamic regulates the stability of target proteins, thereby influencing tumor progression^18,19^. The dynamic interplay between ubiquitination and deubiquitination is linked to several cellular processes, including signal transduction, stress responses, and DNA damage repair, and is implicated in the regulation of tumor progression^20,21^. However, the role of palmitoylation and ubiquitination of key proteins in GCLM remains underexplored, necessitating a thorough and detailed investigation.

Ferroptosis is an iron-dependent form of cell death distinct from autophagy and apoptosis in terms of morphology, genetics, and biochemistry^22,23^. The hallmark of ferroptosis is oxidative damage within the cell, driven by lipid peroxidation and the excessive accumulation of iron-induced reactive oxygen species (ROS). This process is closely linked to the depletion of glutathione (GSH) and the inactivation of glutathione peroxidase 4 (GPX4) and solute carrier family 7 member 11 (SLC7A11), ultimately leading to the buildup of iron-dependent lipid peroxides^24,25^. Numerous studies have demonstrated that ferroptosis acts as a natural tumor-suppressive mechanism, playing a critical role in regulating tumor growth. It has been implicated in the development and therapeutic response of several cancers, including colorectal cancer (CRC), lung cancer, pancreatic cancer, and breast cancer^26,27^. SLC7A11, a key component of cysteine transporters, is increasingly recognized for its role in regulating tumor ferroptosis^28^. For instance, in CRC, LPCAT2 has been shown to induce ferroptosis by inhibiting PRMT1 nuclear translocation, thereby diminishing PRMT1’s positive regulation of the SLC7A11 promoter^29^. Another study reported that APE1 regulates ferroptosis in hepatocellular carcinoma (HCC) by modulating the redox activity of the NRF2/SLC7A11/GPX4 axis. Inhibition of APE1, both genetically and chemically, promotes ubiquitin-proteasome-dependent degradation of NRF2, subsequently suppressing SLC7A11 and GPX4 expression and leading to ferroptosis in HCC cells^30^. Despite these findings, the relationship between molecules that promote GCLM and ferroptosis remains unexplored.

In this study, ASF1B was identified as a key promoter of GCLM through label-free proteomics. Subsequent experiments revealed that ferroptosis and ZDHHC9 are implicated in ASF1B’s promotion of GCLM. The results also indicate a molecular interaction between ZDHHC9 and PCBP1. Mechanistically, ZDHHC9 palmitoylates PCBP1 at the C109 residue, thereby inhibiting its ubiquitination and stabilizing its expression. PCBP1, in turn, upregulates SLC7A11 by stabilizing its mRNA, inhibiting ferroptosis and synergizing with ZDHHC9. Collectively, this study uncovers novel roles and mechanisms of SLC7A11 in promoting ASF1B-mediated GCLM, underscoring the potential of targeting the ASF1B/ZDHHC9/PCBP1 axis as a therapeutic strategy for GCLM.

## Results

### ASF1B expression is upregulated in GCLM tissues and associated with unfavorable prognosis

**Fig. 1:**
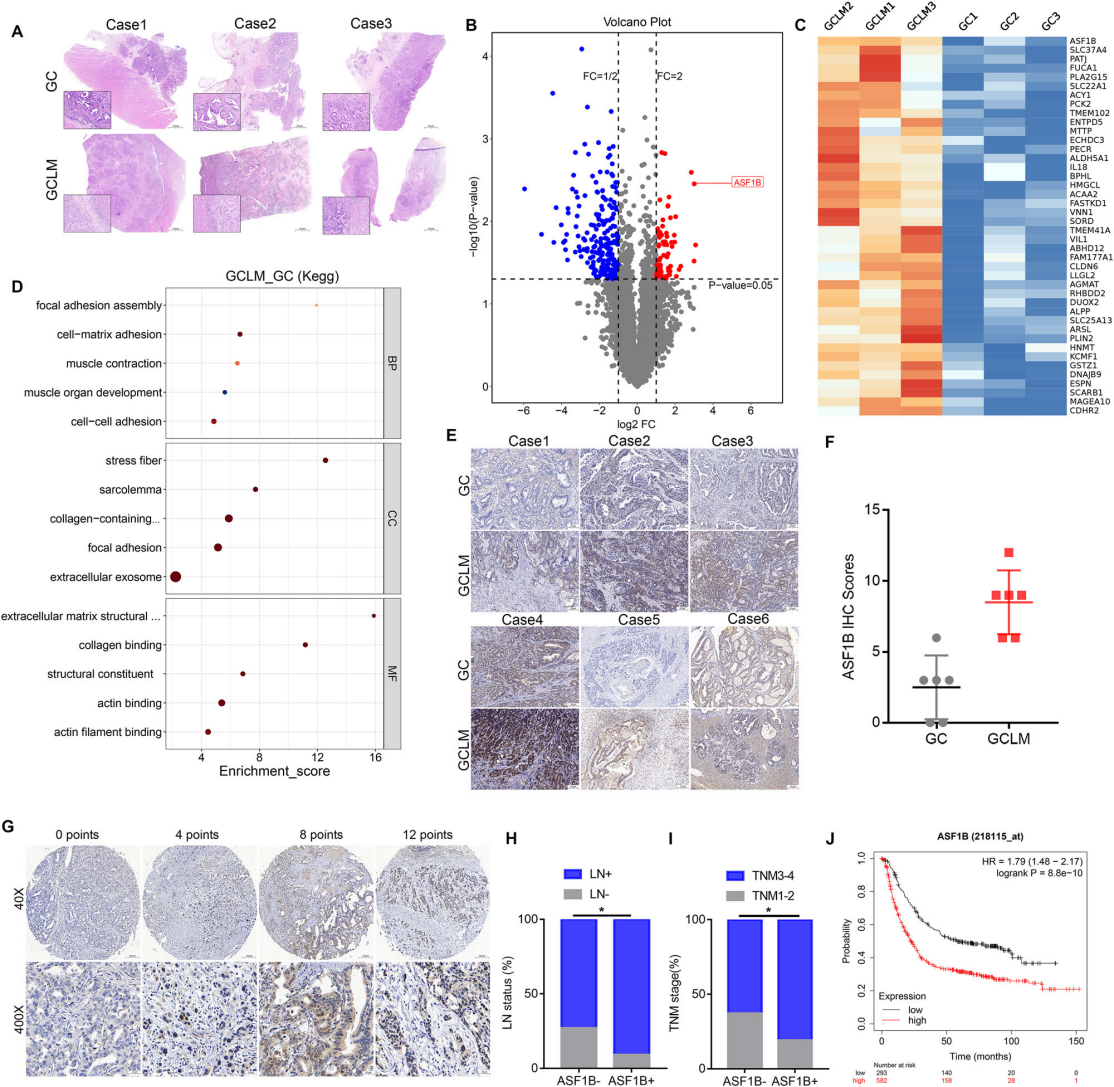
ASF1B expression is upregulated in GCLM tissues and associated with unfavorable prognosis. **A** Panoramic Hematoxylin and Eosin staining of three GCLM patients for label-free proteomics analysis. **B**, **C** Label-free proteomics analysis of differentially expressed proteins (DEPs, DEPs defined by |Log₂FC | > 2 and P <0.05) and their display in volcano and heat maps, ASF1B was used as a significantly differentially expressed protein for further research. **D** Gene enrichment analysis suggests that the differences between liver metastases and primary lesions were mainly concentrated in pathways related to cell adhesion and cell metastasis. **E** Immunohistochemical staining of six pairs of GCLM tissue sections showed increased expression of ASF1B in liver metastasis tissues. **F** The immunohistochemical score of AFS1B in the GCLM group was significantly higher than that in the GC primary lesion group. **G** TMA immunohistochemical staining showed an increase in the staining intensity of ASF1B in GC tissue. **H**, **I** Immunohistochemical score indicated that ASF1B expression score was correlated with lymph node positivity and tumor stage. **J** Kaplan-Meier analysis suggested that ASF1B expression was significantly correlated with prognosis of GC patients.

Three pairs of tissue sections from GCLM were initially selected for analysis, with panoramic staining confirming the presence of liver metastasis (Fig. 1A). The dissolved proteins from these GCLM tissue sections were then subjected to label-free proteomics analysis. The resulting volcano plot revealed that, compared to the primary GC lesion, 71 proteins were upregulated and 213 were downregulated in the liver metastasis group (Fig. 1B). Notably, heatmap analysis indicated that ASF1B exhibited the most significant differential expression among the upregulated proteins (Fig. 1C). The minimal variation observed between the three pairs of GCLM tissue samples further supported the reliability of these findings (Supplementary Fig. S1A-B). Additionally, the high proportion of proteins containing more than 15 peptide segments underscored the confidence in protein identification (Supplementary Fig. S1C). KEGG enrichment analysis revealed that the gene set highly expressed in liver metastatic tissues was functionally associated with cell adhesion and local lesion adhesion, emphasizing ASF1B’s potential role in driving GCLM (Fig.1D). Immunohistochemistry also showed a significant increase in ASF1B expression in liver metastatic tissues (Fig. 1E-F). Furthermore, ASF1B expression was evaluated on a TMA containing 107 GC tissues and 22 adjacent normal tissues. Representative images displayed varying levels of ASF1B expression, with a marked increase observed in GC tissues (Fig. 1G). This observation was further corroborated by the UALCAN database, which confirmed significantly higher ASF1B mRNA expression in GC tissues compared to normal tissues (Supplementary Fig. S1D). To elucidate the clinical significance of ASF1B in GC, the relationship between ASF1B expression and clinicopathological features was analyzed. Results demonstrated that patients with positive ASF1B expression exhibited higher rates of lymph node positivity and advanced TNM staging compared to those with negative ASF1B expression (Fig. 1H-I). Moreover, Kaplan-Meier analysis revealed that patients with GC exhibiting elevated ASF1B expression had significantly shorter survival times (Fig. 1J). Collectively, these results suggest that ASF1B is upregulated in GCLM tissues and is correlated with advanced clinicopathological features and poor prognosis in GC.

### Overexpression of ASF1B promotes GCLM and malignant phenotype of GC cells

**Fig. 2:**
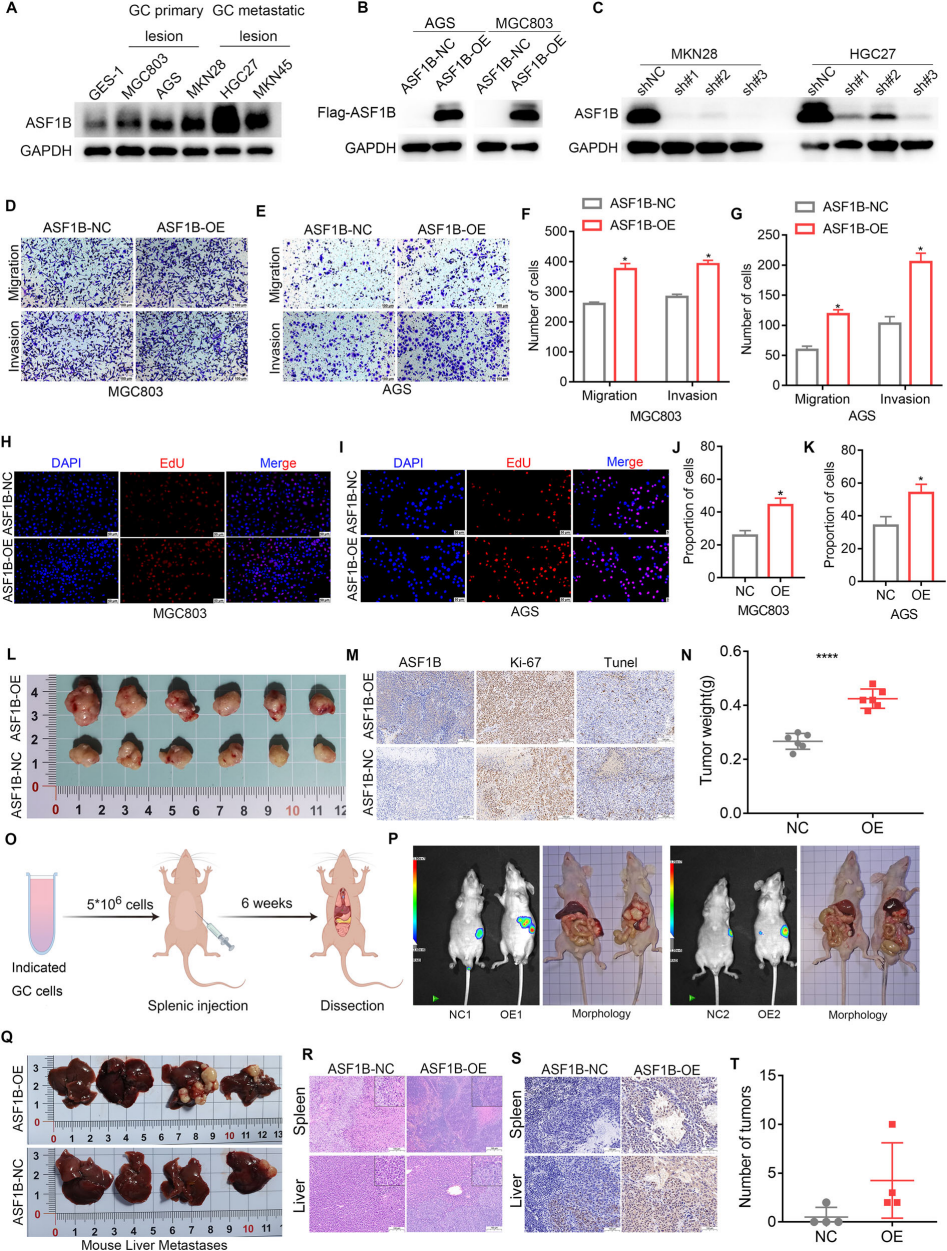
Overexpression of ASF1B promotes GCLM and malignant phenotype of GC cells. **A** Western blot showed that ASF1B protein expression was significantly up-regulated in GC cell lines. Compared to the cell lines (MGC803 and AGS) derive from the primary GC lesions, the expression level of ASF1B was significantly increased in the cell lines (MKN28, HGC27, and MKN45) derive from the GC metastasis lesions. **B**, **C** The overexpression and knockdown efficiency of ASF1B were confirmed by Western blot analysis, sh#1 and sh#3 showed a better knockdown efficiency. **D–G** Transwell assay showed that overexpression of ASF1B significantly increased the invasion and migration ability of MGC803 and AGS cells. **H–K** EdU analysis demonstrated that overexpression of ASF1B can significantly enhance the cell proliferation of MGC803 and AGS cells. **L** Overexpression of ASF1B significantly increases subcutaneous tumor volume in nude mice. **M** Ki-67 and Tunel staining showed that overexpression of ASF1B increased tumor cell proliferation and decreased apoptosis ability. **N** Overexpression of ASF1B significantly increases subcutaneous tumor weight in nude mice. **O** Procedures of splenic injection for GCLM. **P** Thermal imaging and morphology of GCLM model constructed by injecting overexpressing ASF1B cells into the spleen. **Q** Mouse GCLM model proves that overexpression of ASF1B increases the number of liver metastases. **R** HE staining of GCLM model constructed by injecting overexpressing ASF1B cells into the spleen. **S** Immunohistochemical staining showed increased expression of ASF1B in the liver of overexpressing mice. **T** Overexpression of ASF1B significantly increases the number of liver metastatic tumor nodules.

To further validate that ASF1B does indeed promote GCLM, lentiviral constructs for ASF1B overexpression and knockdown were generated for functional assays. Initial validation of ASF1B protein expression in GC cell lines revealed that ASF1B levels were elevated in GC cells compared to GES-1. Notably, ASF1B expression was significantly higher in cell lines derived from metastatic GC lesions (MKN28, HGC27, and MKN45) than in those from primary GC lesions (MGC803 and AGS) (Fig. 2A; Supplementary Fig. S2A). Based on these expression patterns, ASF1B was overexpressed in AGS and MGC803 cells, while ASF1B was knocked down in MKN28 and HGC27 cells. The efficiency of lentiviral infection was confirmed via western blot, with sh#1 and sh#3 selected for subsequent experiments (Fig. 2B, C). ASF1B overexpression markedly enhanced the migration and invasion capabilities of MGC803 and AGS cells, whereas ASF1B knockdown significantly reduced these abilities in MKN28 and HGC27 cells (Fig. 2D–G; Supplementary Fig. S2B–E). EDU assays further demonstrated that ASF1B overexpression substantially increased the proliferation of MGC803 and AGS cells, while ASF1B silencing diminished the proliferative capacity of MKN28 and HGC27 cells (Fig. 2H–K; Supplementary Fig. S2F–I). Subcutaneous xenograft confirmed that overexpression of ASF1B significantly increased the volume and weight of subcutaneous tumors compared to the control group (Fig. 2L, N). Immunohistochemical results showed that overexpression of ASF1B led to an increase in Ki-67 staining within the tumor. Tunel staining showed a decrease in the apoptotic ability of tumor cells after overexpression of ASF1B (Fig. 2M). Following in vitro and in vivo confirmation of ASF1B’s role in promoting the malignant progression of GC, its ability to enhance GCLM was further validated in mouse models (Fig. 2O). Thermal imaging and morphological analysis (Fig. 2P) revealed that mice injected with ASF1B-overexpressing GC cells developed metastatic liver tumors, with tumor thermography also detected in the spleens of both experimental groups. Dissection of the liver metastasis model revealed that 25% (1/4) of control mice developed liver tumors, whereas all mice overexpressing ASF1B exhibited liver tumor nodules (4/4) and HE staining confirmed the presence of tumors in both the liver and spleen (Fig. 2Q, R). Immunohistochemical of liver tissues further demonstrated higher ASF1B staining intensity in the overexpression group compared to controls and the number of liver metastatic tumor nodules significantly increased after ASF1B overexpression (Fig. 2S-T). These findings indicate that ASF1B significantly promotes the malignant progression of GC and enhances GCLM.

### Knocking down ASF1B increases SLC7A11-mediated ferroptosis levels in GC cells

**Fig. 3:**
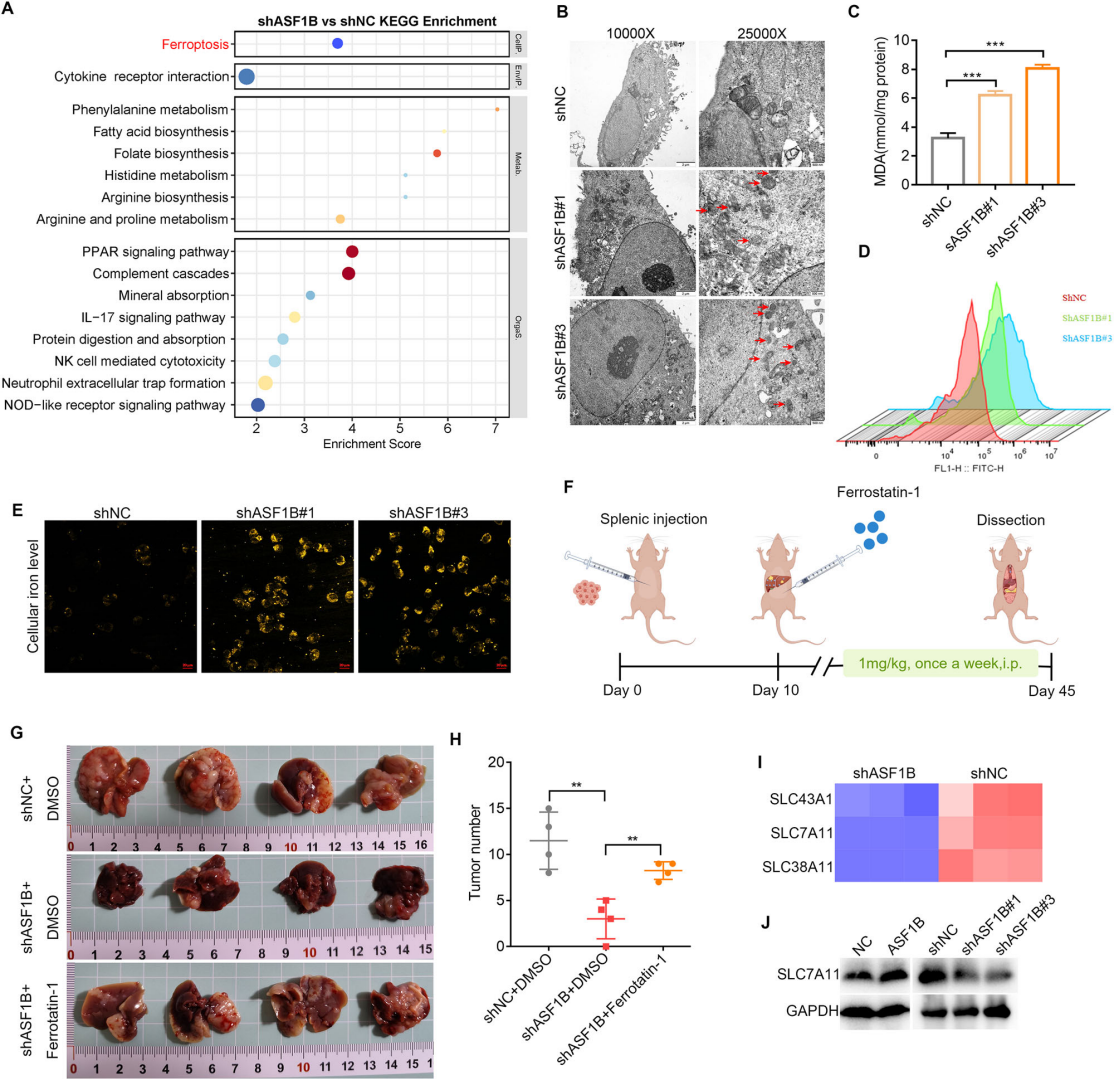
Knocking down ASF1B increases SLC7A11-mediated ferroptosis levels in GC cells. **A** KEGG enrichment analysis indicates that knocking down of ASF1B is associated with ferroptosis related pathway. **B** Transmission electron microscopy showed that ASF1B knockdown caused mitochondrial atrophy, which reconfirmed the results of RNA sequencing. **C–E** Knocking down ASF1B leads to an increase in MDA, ROS and cellular iron levels. **F–H** Compared with the control group, the ASF1B knockdown group showed a significant reduction in liver metastasis and the number of metastatic nodules, and Fer-1 reversed this inhibitory effect. **I**, **J** Heatmap and WB shows correlation between ASF1B and SLC7A11 expression.

To elucidate the mechanism by which ASF1B promotes GCLM, RNA sequencing was performed following ASF1B knockdown. KEGG analysis showed that after knocking down ASF1B, the metabolic pathway of GC cells was enriched in ferroptosis (Fig. 3A). TEM further corroborated these outcomes, as ASF1B knockdown resulted in mitochondrial atrophy (Fig. 3B). The MDA, ROS and cellular iron level detection results also showed that knocking down ASF1B significantly increased the level of ferroptosis in GC cells (Fig. 3C–E). Subsequently, the effect of ferroptosis levels on the GCLM was further validated in vivo. The ferroptosis level in nude mice was altered by intraperitoneal injection of the ferroptosis inhibitor Fer-1 (Fig. 3F). The results showed that knocking down ASF1B significantly reduced the liver metastasis ability of GC, while injecting Fer-1 in vivo reversed this inhibitory effect (Fig. 3G, H). The heat map further showed that knocking down ASF1B significantly reduced the expression of SLC7A11, a key marker protein for ferroptosis (Fig. 3I). Meanwhile, western blot showed that overexpression of ASF1B significantly increased the expression of SLC7A11, while ASF1B knockdown significantly reduced its protein levels (Fig. 3J). These results suggest that ASF1B can regulate SLC7A11-mediated ferroptosis, thereby promoting GCLM.

### ASF1B upregulates ZDHHC9 transcription level via HOXB3, thereby inhibiting SLC7A11-ferroptosis

**Fig. 4:**
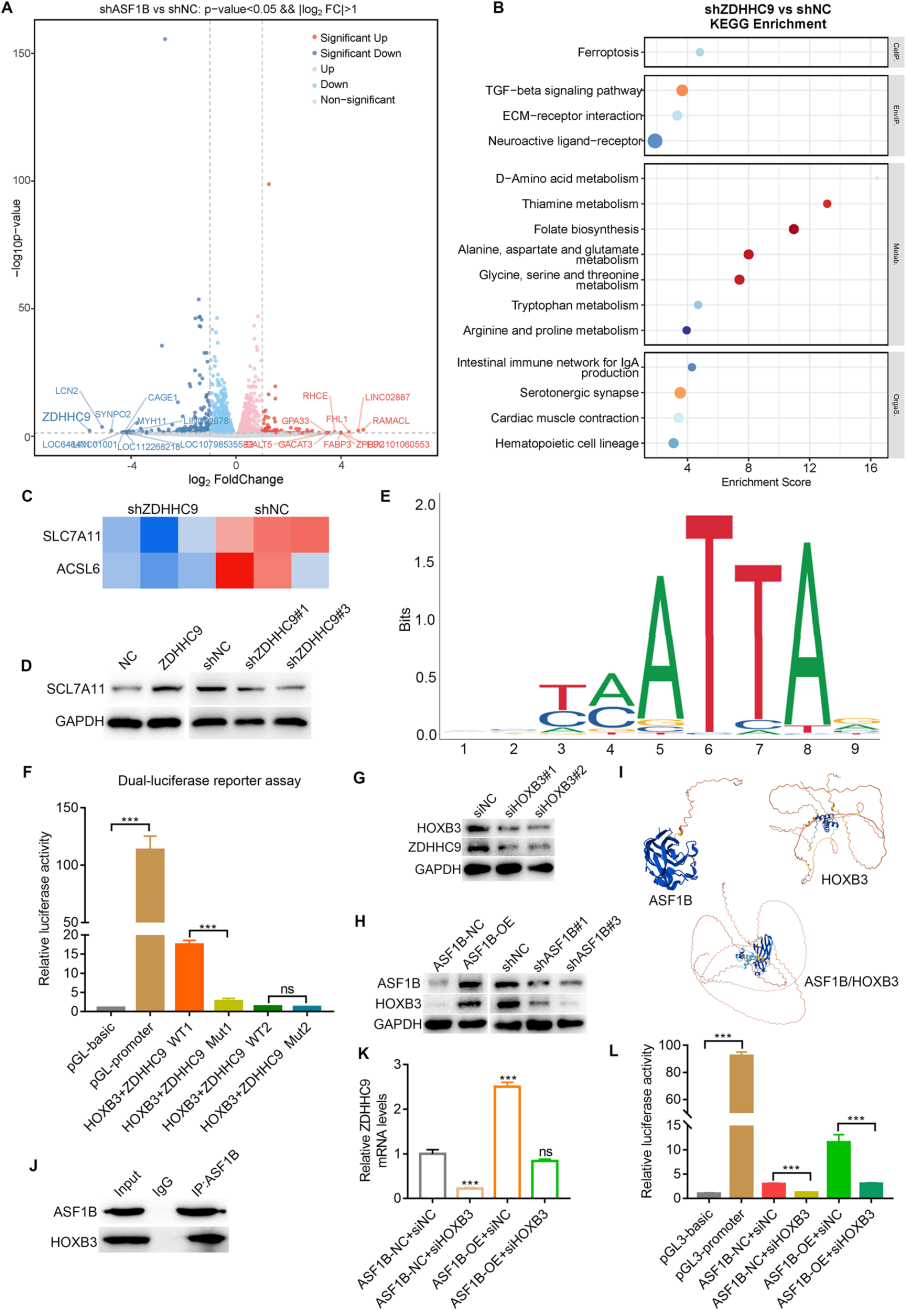
ASF1B upregulates ZDHHC9 transcription level via HOXB3, thereby inhibiting SLC7A11-ferroptosis. **A** RNA sequencing after ASF1B knocking down shows a correlation between ASF1B and ZDHHC9 expression. **B** RNA sequencing after ZDHHC9 knocking down shows that differentially expressed genes enriched in the ferroptosis metabolic pathway. **C**, **D** ZDHHC9 regulates mRNA and protein expression of SLC7A11. **E** JASPAR predicts the binding site of HOXB3 and ZDHHC9 promoter. **F** Dual luciferase assay suggested that transcription factor HOXB3 could bind to the promoter site of ZDHHC9, and the luciferase activity was weakened after mutation of the promoter site. **G**, **H** Immunoblotting results confirmed that ASF1B could regulate the expression of ZDHHC9 through transcription factor HOXB3. **I** Alphafold’s protein docking analysis showed a molecular connection between ASF1B and HOXB3. **J** Co-immunoprecipitation indicates that HOXB3 binds to ASF1B. **K**, **L** RT-PCR and luciferase assays further confirmed that ASF1B upregulates ZDHHC9 transcription via HOXB3.

In order to further explore the downstream target proteins regulated by ASF1B, volcano and heatmap analysis was performed based on RNA sequencing data, and the results showed that ZDHHC9 may be a key downstream protein (Fig. 4A; Supplementary Fig. S3). After screening ZDHHC9 as a downstream target molecule of ASF1B, RNA sequencing analysis was performed after knocking down ZDHHC9. KEGG results showed that differentially expressed genes were enriched in the ferroptosis pathway, consistent with the enrichment pattern observed after ASF1B knockdown (Fig. 4B). Similarly to ASF1B knockdown, ZDHHC9 can also regulate the protein expression of SLC7A11 (Fig. 4C, D). To explore the mechanism of ASF1B regulating ZDHHC9 expression, dual luciferase assay was conducted. The results suggested that transcription factor HOXB3 could bind to the promoter site of ZDHHC9, and the luciferase activity was weakened after mutation of the promoter site (Fig. 4E, F). Immunoblotting results further confirmed that ASF1B could upregulate the expression of ZDHHC9 through HOXB3 (Fig. 4G, H). Subsequently, AlphaFold protein docking analysis and co-immunoprecipitation (Co-IP) assays suggested a potential interaction between ASF1B and HOXB3 (Fig. 4I, J). Follow-up RT-PCR and luciferase assays further confirmed that ASF1B upregulates ZDHHC9 transcription via HOXB3 (Fig. 4K, L).

### ZDHHC9 promotes the proliferation and metastasis ability of GC in vitro and in vivo

**Fig. 5:**
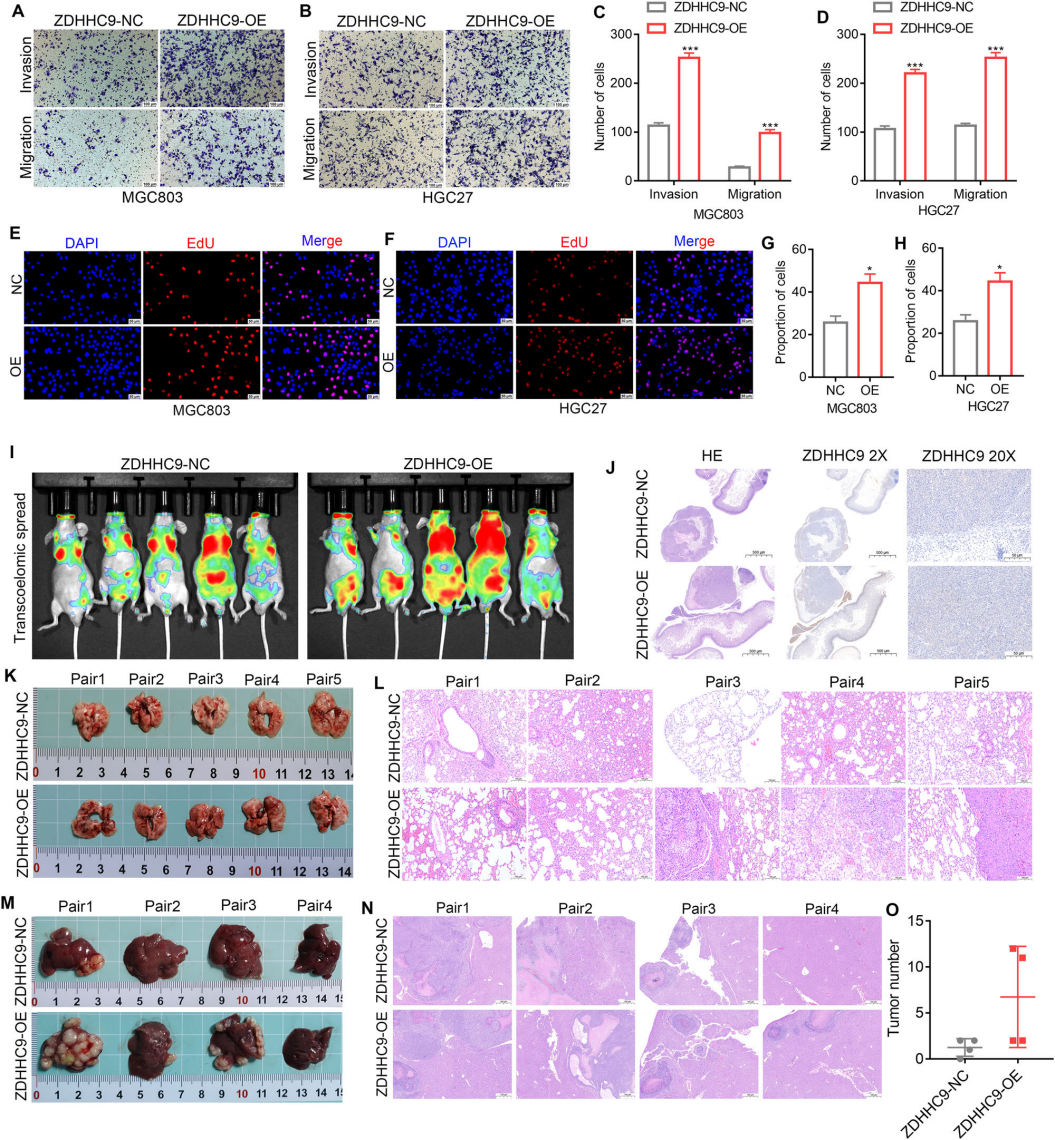
ZDHHC9 promotes the proliferation and metastasis ability of GC in vitro and in vivo. **A–D** Transwell assays indicated that overexpression of ZDHHC9 significantly enhances the migration and invasion characteristics of HGC27 cells, and it was inhibited after silencing ZDHHC9. **E–H** EDU results showed that overexpression of ZDHHC9 increased cell proliferation. **I**, **J** Overexpression of ZDHHC9 leads to wider peritoneal metastasis. **K**, **L** After injecting overexpressed ZDHHC9 into the tail vein, lung metastasis occurred, while the control group did not show lung metastasis. **M–O** Overexpression of ZDHHC9 significantly increased the number of GCLM nodules in nude mouse models.

Then the impact of ZDHHC9 knockdown and overexpression on the malignant phenotype of GC cells was further investigated. As depicted in Fig. 5A–D and Supplementary Fig. S4A–D, ZDHHC9 overexpression markedly enhanced the migration and invasion properties of GC cells. Conversely, ZDHHC9 knockout significantly reduced the migration and invasion capabilities of GC cells. Moreover, EDU assays revealed that ZDHHC9 overexpression promoted cell proliferation, while its silencing inhibited proliferation (Fig. 5E–H; Supplementary Fig. S4E–H). Flow cytometry analysis demonstrated that ZDHHC9 silencing increased apoptosis and inhibited cell cycle progression in GC cells (Supplementary Fig. S4I–M). To comprehensively assess the role of ZDHHC9 in GC cell metastasis in vivo, intraperitoneal injection and tail vein injection models were employed. The results from thermal imaging, HE staining, and ZDHHC9 immunohistochemistry indicated more extensive peritoneal metastasis in the ZDHHC9 overexpression group (Fig. 5I, J). Additionally, six weeks after tail vein injection, dissection revealed three metastases in the overexpression group, with none observed in the control group. HE staining confirmed the presence of lung metastases (Fig. 5K, L; Supplementary Fig. S4N). Furthermore, compared with the control group, overexpression of ZDHHC9 significantly increased the number of GC liver metastatic tumor nodules, and the presence of metastatic tumors was confirmed by HE staining (Fig. 5M–O). Collectively, these results demonstrate that ZDHHC9 significantly enhances the proliferation and metastatic potential of GC both in vitro and in vivo.

### ZDHHC9 palmitoylates PCBP1 at Cys109 residue to inhibit its ubiquitination and degradation

**Fig. 6:**
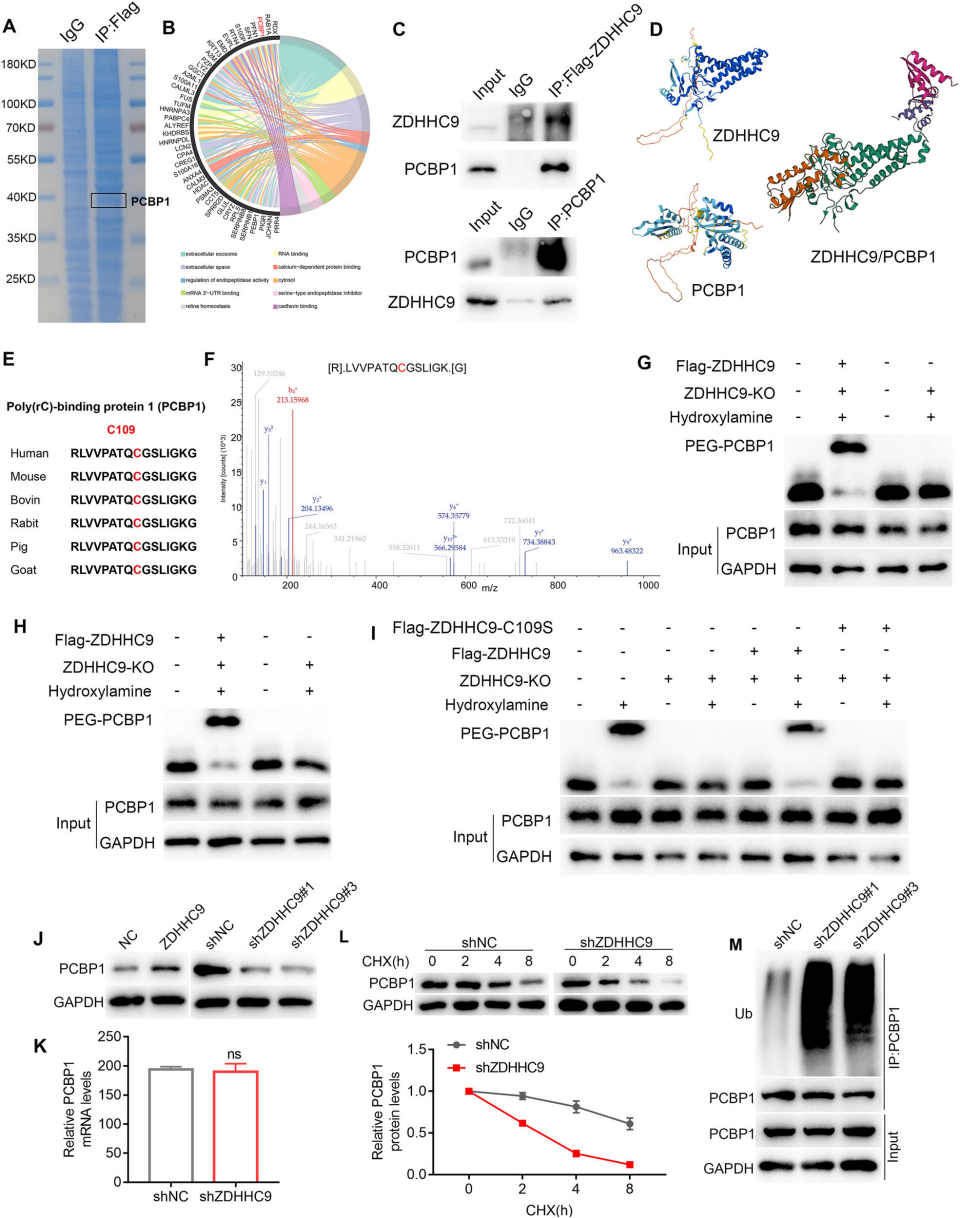
ZDHHC9 promote the occurrence of GCLM through palmitoylating PCBP1 at Cys109 site. **A**, **B** Coomassie blue staining showed that the protein bound to ZDHHC9 was pulled down, and the binding between PCBP1 and ZDHHC9 may be high. **C** Immunoprecipitation indicates that PCBP1 binds to ZDHHC9. **D** Alphafold’s protein docking analysis also showed a molecular connection between ZDHHC9 and PCBP1. **E**, **F** The secondary mass spectrum indicates that the peptide segment of ZHDDC9 binding to PCBP1 includes the palmitoylation site Cys109. **G**, **H** ABE results confirmed that ZDHHC9 can palmitoylate PCBP1, PEG: polyethylene glycol. **I** Mutating PCBP1-Cys109 to serine eliminated the palmitoylation effect of ZDHHC9 on PCBP1. **J** Overexpression or silencing of ZDHHC9 could promote or inhibit the expression of PCBP1. **K** The RNA expression of PCBP1 remained unchanged after silencing ZDHHC9. **L** An increasing protein degradation of PCBP1 was detected using CHX assay after ZDHHC9 knockdown. **M** After silencing ZDHHC9, the ubiquitination of PCBP1 increases.

**Fig. 7:**
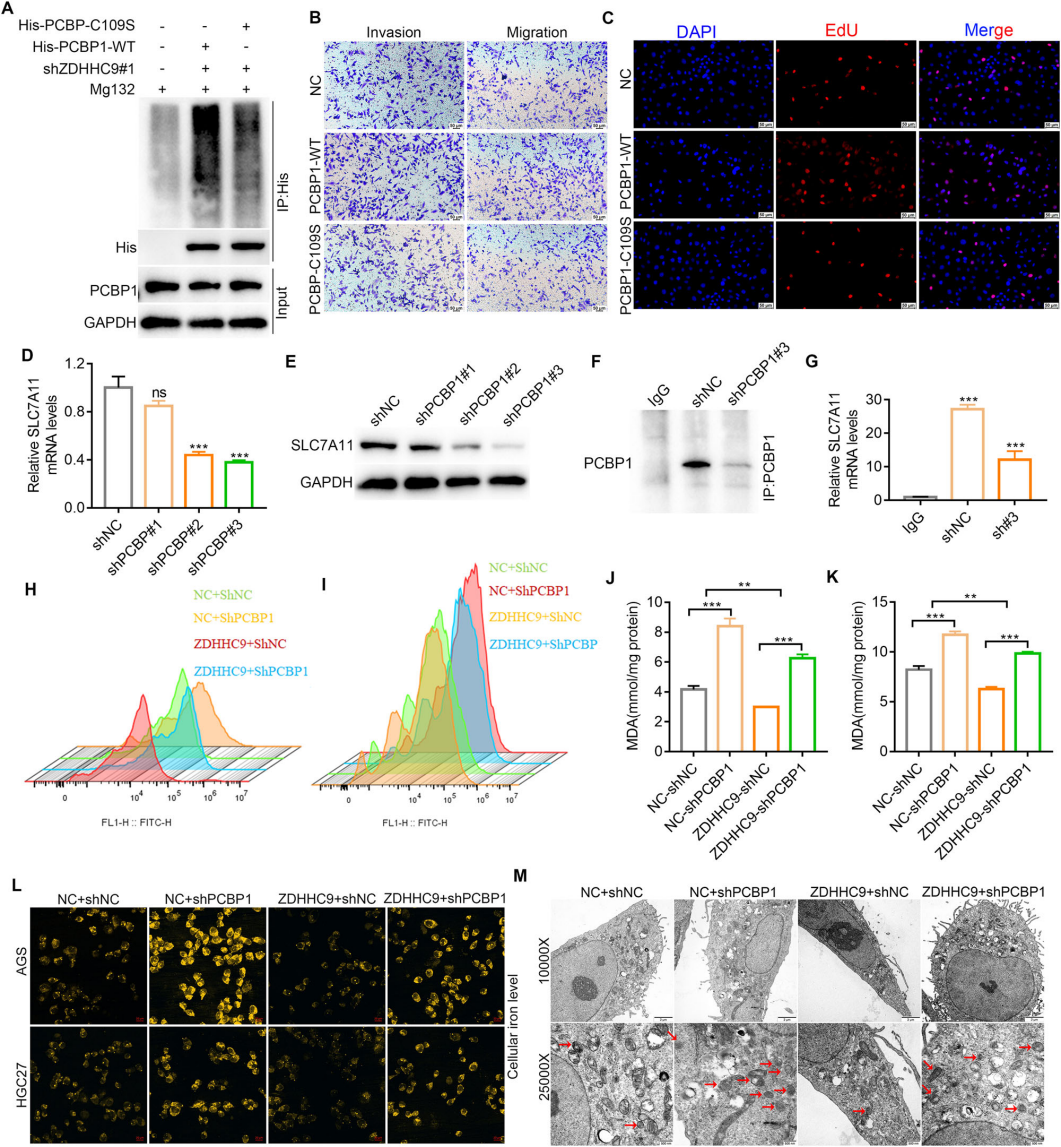
Knocking down PCBP1 reverses ferroptosis inhibition induced by overexpression of ZDHHC9. **A** Mutating PCBP1-Cys109 to serine reversed the upregulation of PCBP1 ubiquitination induced by ZDHHC9 silencing. **B** Transwell assays confirmed that mutation of the PCBP Cys109 residue significantly enhances the invasion and metastasis capabilities of GC cells. **C** EdU assay confirmed that mutation of the PCBP Cys109 residue significantly enhances proliferative capacity of GC cells. **D**, **E** Knocking down PCBP1 significantly reduces the expression of SLC7A11 at both RNA and protein levels. **F** Immunoprecipitation reveals that PCBP1 was successfully pulled down before RIP assay. **G** RIP assay shows that PCBP1 knockdown leads to a decrease in the level of SLC7A11 mRNA. **H–L** ROS, MDA and intracellular iron ion levels detection showed that knocking out PCBP1 increased ferroptosis levels. **L** Observation of mitochondrial morphology in ZDHHC9 overexpressing and PCBP1 knocking down cells using transmission electron microscopy.

To further elucidate the molecular mechanisms by which ZDHHC9 promotes GCLM and identify its potential palmitoyltransferase targets, IP-MS analysis was conducted following ZDHHC9 overexpression. Coomassie blue staining revealed that ZDHHC9 protein position on the pulled-down Flag tape, suggesting the presence of ZDHHC9-interacting proteins, including PCBP1 (Fig. 6A-B). To validate the IP-MS results, co-immunoprecipitation was performed, confirming the interaction between ZDHHC9 and PCBP1 (Fig. 6C). Alphafold’s protein docking analysis showed a molecular connection between ZDHHC9 and PCBP1(Fig. 5D). Immunofluorescence also showed that PCBP1 and ZDHHC9 are co-localized in GC tissues (Supplementary Fig. S5A). Palmitoylation involves the reversible attachment of palmitoyl groups to cysteine residues in proteins. Using the Swisspalm palmitoylation prediction tool (https://swisspalm.org), three potential palmitoylation sites in PCBP1 were identified: Cys109, Cys54, and Cys158 (Supplementary Fig. S5B). Further analysis of the secondary mass spectrum from the IP-MS revealed that the peptide segment binding ZDHHC9 to PCBP1 includes the palmitoylation site Cys109, strongly suggesting that ZDHHC9 palmitoylates PCBP1 (Fig. 6F). Then the ABE experiments^31^ were conducted and the results demonstrated that ZDHHC9 knockout in AGS and HGC27 cells abolished PCBP1 palmitoylation by ZDHHC9 (Fig. 6G-H). Furthermore, mutation of PCBP1 at Cys109 to serine also eliminated the palmitoylation effect of ZDHHC9 on PCBP1 (Fig. 6I). Thus, ZDHHC9 promotes GCLM by palmitoylating PCBP1 at Cys109. Validation of their molecular interplay revealed that ZDHHC9 overexpression or knockdown in AGS cells correspondingly upregulated or downregulated PCBP1 protein levels (Fig. 56). Notably, ZDHHC9 silencing did not alter PCBP1 RNA expression, indicating post-translational control of PCBP1 degradation (Fig. 6K). Subsequently, CHX chase assays showed that knocking out ZDHHC9 significantly increased the protein decay rate of PCBP1 (Fig. 6L). Based on prior research that ZDHHC proteins stabilize targets by reducing ubiquitination^32^, we hypothesized ZDHHC9 promotes PCBP1 by inhibiting its ubiquitination. Testing this in ZDHHC9-knockdown cells showed increased PCBP1 ubiquitination versus controls, confirming ZDHHC9 maintains PCBP1 stability via this mechanism (Fig. 6M). Then mutating the PCBP1-Cys109 site to serine demonstrated that the increase in PCBP1 ubiquitination observed after ZDHHC9 silencing was reversed, confirming that ZDHHC9 stabilizes PCBP1 expression by inhibiting its ubiquitination (Fig. 7A). Subsequent cell function assays confirmed that mutation of the PCBP Cys109 residue significantly enhances the invasion and metastasis capabilities of GC cells (Fig. 7B-C).

### Knocking down PCBP1 reverses ferroptosis inhibition induced by overexpression of ZDHHC9

Next, further explore the effect of PCBP1 expression on ferroptosis levels in GC cells. Given that PCBP1, an iron regulatory protein, functions as a key ferroptosis regulator, we investigated its role in GC cell ferroptosis. PCBP1 knockdown significantly reduced SLC7A11 expression at both transcriptional and translational levels (Fig. 7D, E). Next, RNA Immunoprecipitation assay confirmed that knocking down PCBP1 significantly reduced the mRNA stability of SLC7A11 (Fig. 7F-G). In vivo, IHC confirmed that ZDHHC9 can regulate the levels of both PCBP1 and SLC7A11 simultaneously (Supplementary Fig. S6). Then ROS detection indicated that ZDHHC9 overexpression inhibited ferroptosis in GC cells, and this effect was reversed upon PCBP1 knockdown (Fig. 7H, I). MDA and cellular iron level detection mirrored this outcome, with PCBP1 silencing promoting ferroptosis in ZDHHC9-overexpressing cells (Fig. 7J–L). Additionally, TEM showed that PCBP1 knockdown in ZDHHC9-overexpressing cells significantly reduced or eliminated mitochondrial cristae, accompanied by an increase in mitochondrial membrane density (Fig. 7M). These results indicate that PCBP1 knockdown can reverse the ferroptosis inhibition induced by ZDHHC9 overexpression.

### Clinical expression correlation of ASF1B/ZDHHC9/PCBP1/SLC7A11 axis

**Fig. 8:**
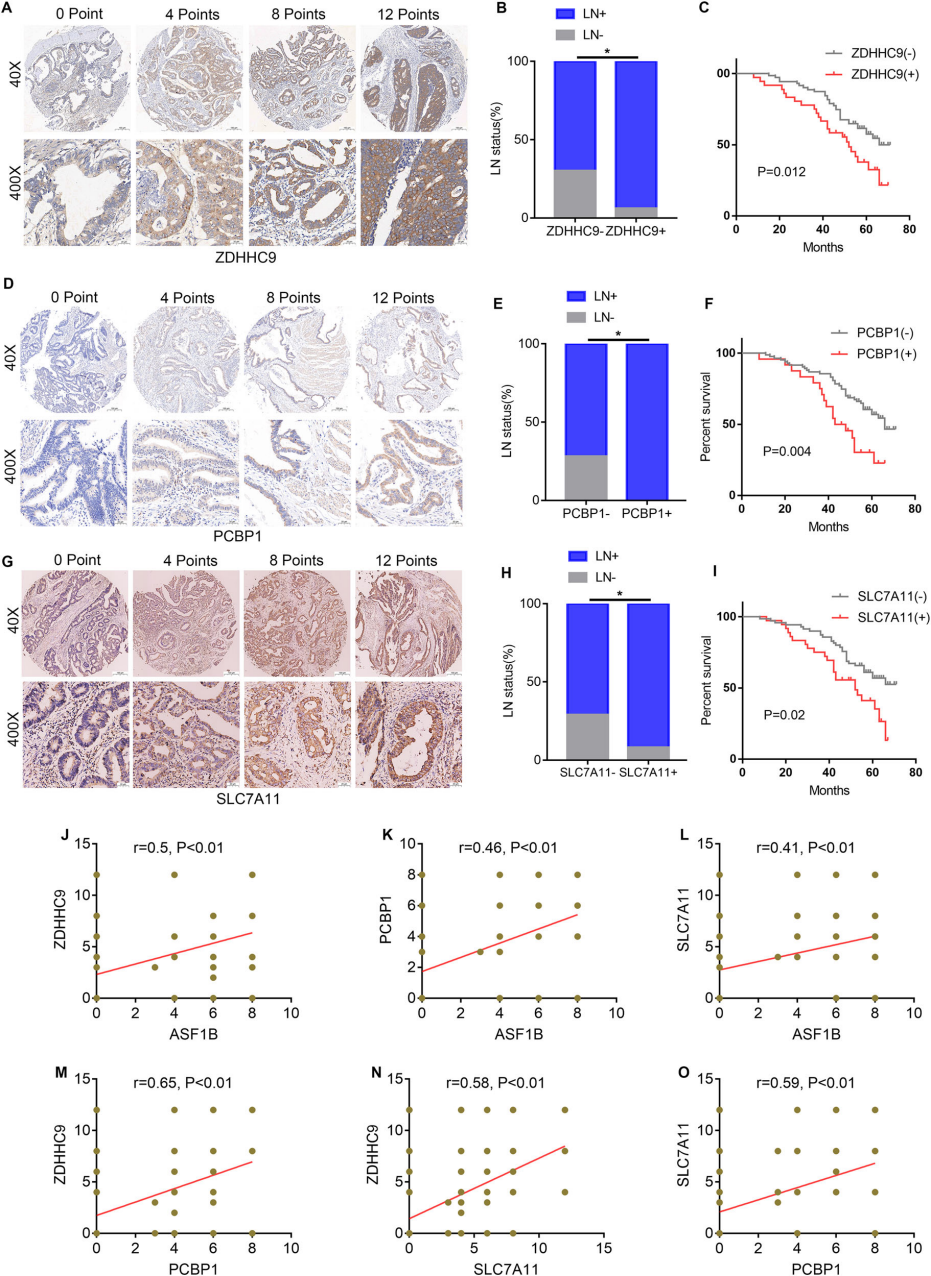
Clinical expression correlation of ASF1B/ZDHHC9/PCBP1/SLC7A11 axis. **A** Representative immunohistochemical staining of ZDHHC9 in TMA. **B** The correlation between ZDHHC9 expression and lymph node positivity rate. **C** Kaplan-Meier survival curve of GC patients with low and high ZDHHC9 expression in tissue chip. **D** Representative immunohistochemical staining of PCBP1 in tissue chip. **E** The correlation between PCBP1 expression and lymph node positivity rate. **F** Kaplan-Meier survival curve of GC patients with low and high PCBP1 expression in tissue chip. **G** Representative immunohistochemical staining of SLC7A11 in tissue chip. **H** The correlation between SLC7A11 expression and lymph node positivity rate. **I** Kaplan-Meier survival curve of GC patients with low and high SLC7A11 expression in tissue chip. **J–O** Immunohistochemical scoring evaluation of the correlation between ASF1B/ZDHHC9/PCBP1/SLC7A11 molecular expression in TMA.

**Fig. 9:**
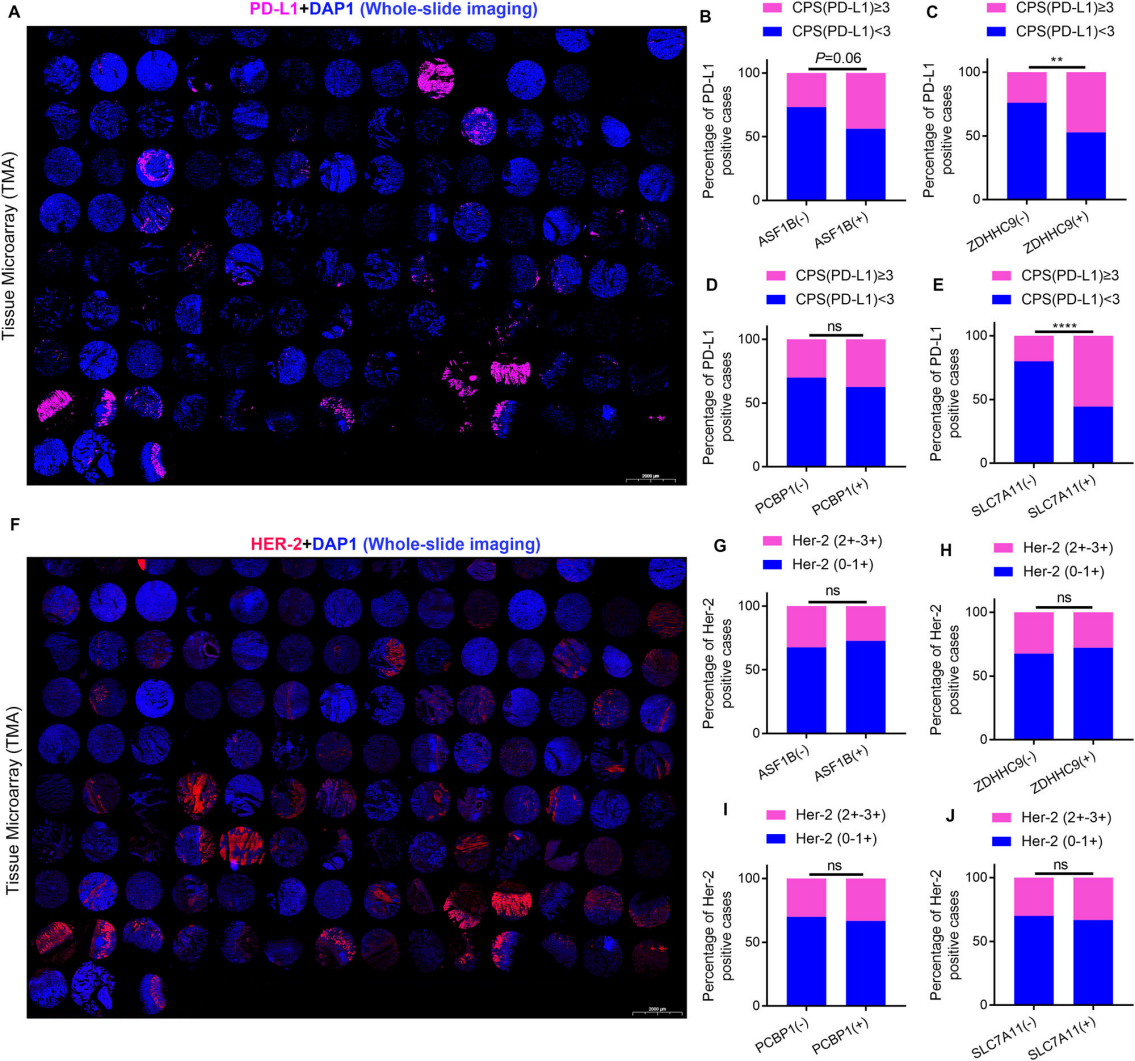
Expression profiles of ASF1B, ZDHHC9, PCBP1, and SLC7A11 across different molecular subtypes of GC. **A**Exhibition of PD-L1 expression in tissue microarrays from the same batch. **B** Chi-square test indicates that a correlation between ASF1B and PD-L1 expression, but the difference was not statistically significant (P = 0.06). **C–E** ZDHHC9 and SLC7A11 were significantly correlated with PD-L1 expression. However, the expression of PCBP1 is not associated with that of PD-L1. **F** Exhibition of Her-2 expression in tissue microarrays from the same batch. **G–J** The ASF1B/ZDHHC9/PCBP1/SLC7A11 signaling axis had no association with HER-2 expression.

TMAs staining showed ZDHHC9 was predominantly membrane-localized, with significantly higher expression in GC than adjacent normal tissues (Fig. 8A). Statistical analysis revealed high ZDHHC9 expression correlated with higher lymph node positivity (Fig. 8B). Survival analysis of 107 GC patients stratified by ZDHHC9 staining showed high expression associated with poorer prognosis (Fig. 8C). Similarly, IHC demonstrated PCBP1 and SLC7A11 were significantly upregulated in GC (Figure 8D, G); their high expression correlated with higher lymph node positivity (Fig. 8E, H) and poorer prognosis (Fig. 8F, I). Correlation analysis by IHC scoring revealed ASF1B expression in GC significantly positively correlated with ZDHHC9, PCBP1, and SLC7A11 (Fig. 8J–L). Additionally, ZDHHC9 positively correlated with PCBP1 and SLC7A11 (Fig. 8M, N), and PCBP1 positively correlated with SLC7A11 (Fig. 8O). Additionally, we detected PD-L1 and HER-2 expression using the same batch of TMAs (Fig. 9A–J). Stratified analysis showed a correlation between ASF1B and PD-L1 expression, but the difference was not statistically significant (Fig. 9B). ZDHHC9 and SLC7A11 were significantly correlated with PD-L1 expression (Fig. 9C, E), while this signaling axis had no association with HER-2 expression (Fig. 9G–J). These results may provide some guidance for immunotherapy in patients with advanced gastric cancer.

## Discussion

**Fig. 10:**
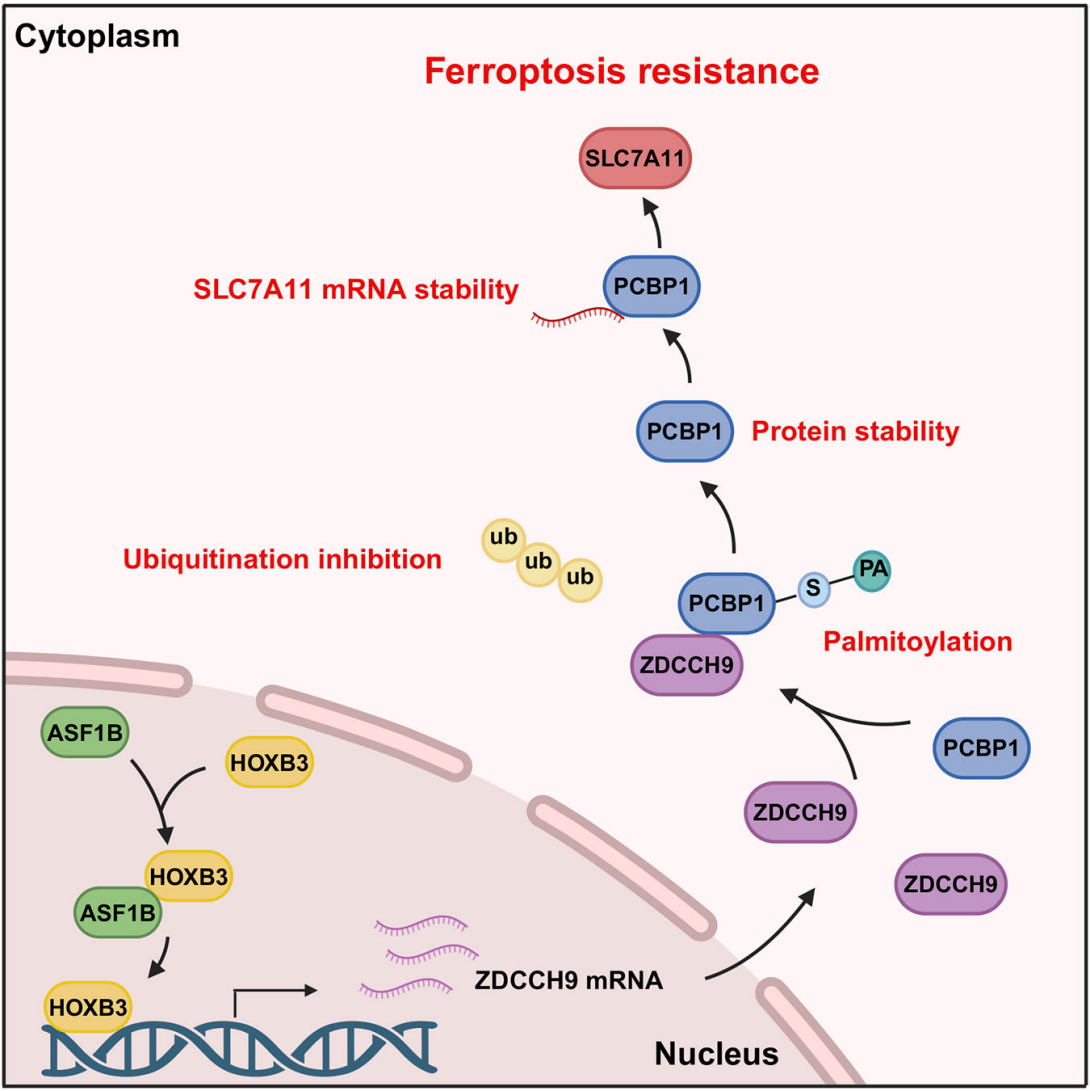
Schematic diagram of the model proposed in this study. ASF1B is upregulated in GCLM and promotes transcription of ZDHHC9 through HOXB3, which stabilizes PCBP1 expression by palmitoylation at the Cys109 site and inhibiting its ubiquitination degradation. ZDHHC9 synergizes with PCBP1 to promote stable expression of SLC7A11 and produce ferroptosis inhibition properties.

The presence of GCLM introduces significant challenges to existing treatment strategies. Conventional surgical resection combined with chemotherapy appears insufficient in effectively countering the molecular mechanisms driving liver metastasis. Therefore, it is imperative to elucidate the underlying molecular mechanisms of GCLM and develop targeted therapeutic strategies. This study clarifies the detailed mechanism by which ASF1B promotes GCLM, providing a partial theoretical basis for the treatment of GCLM (Fig. 10). In recent years, research on GCLM has been advancing rapidly. For example, studies in areas such as tumor-associated macrophages, natural killer cells, and ferroptosis have all indicated potential links to GCLM^24,33,34^. Nevertheless, ASF1B, a member of the ASF1 family and the focus of this study, may offer a new research direction for exploring the mechanism of gastric cancer liver metastasis. Within the ASF1 family, ASF1B and ASF1A share a conserved core histone H3-H4 binding domain but exhibit distinct functions^35^. ASF1A primarily participates in DNA repair and regulates cellular aging, whereas ASF1B predominantly influences cell cycle progression and proliferation rate^36^. Additionally, ASF1B impacts the tumor microenvironment by promoting immune cell infiltration^37^. However, the association between ASF1B and GCLM remains unclear. Based on label-free proteomics analysis of clinical samples, we innovatively proposed that ASF1B is associated with GCLM. Furthermore, through rigorous experimental research, we identified the ASF1B/ZDHHC9/PCBP1/SLC7A11 signaling axis. This axis ultimately fills the research gap in the crosstalk mechanism between epigenetic regulation and ferroptosis in GCLM.

As a palmitoyltransferase, ZDHHC9 serves as a pivotal molecule in the present study. First, the interaction between ZDHHC9 and PCBP1 entails the crosstalk between palmitoylation and ubiquitination regulatory cascades. Second, ZDHHC9 maintains a close association with ferroptosis, mediated by PCBP1 and SLC7A11. These aforementioned mechanisms represent the core regulatory axes interrogated in this research. We will initially deliberate on the crosstalk governing the coordinated regulation of palmitoylation and ubiquitination, followed by an investigation into the universality of the association between ZDHHC9-mediated protein palmitoylation and ferroptosis. In hepatocellular carcinoma, palmitoylation of PHF2 mediated by ZDHHC23 promotes the ubiquitination-dependent degradation of PHF2. In turn, PHF2 directly reduces the stability of SREBP1c, thereby decreasing SREBP1c-regulated lipogenesis^38^. The cytoplasmic domain of PD-L1 is also subject to palmitoylation modification. Studies have shown that PD-L1 can be palmitoylated by ZDHHC3, which inhibits the ubiquitination-dependent degradation of PD-L1 and thereby enhances the immune killing effect of T cells against tumors^32,39^. In the present study, we also found a significant correlation between the expression of PD-L1 and ZDHHC9, suggesting that the ZDHHC family may have a potential close association with tumor immunotherapy. The aforementioned authoritative studies all indicate that there is a close association between palmitoylation and protein ubiquitination, and this crosstalk plays an extremely important role in tumor progression.

Ferroptosis, an iron-dependent form of cell death, remains incompletely understood in the context of its association with GCLM. PCBP1, a multifunctional RNA-binding protein, plays a critical role in gene transcription and RNA regulation^40^. As a cytoplasmic iron-binding partner, PCBP1 is also implicated in the regulation of intracellular ferroptosis levels^41^. Recent research in head and neck cancer demonstrated that PCBP1 knockout facilitates polyunsaturated fatty acid peroxidation by upregulating ALOX15 expression. Additionally, excessive iron accumulation counteracts PCBP1’s inhibitory effect on mitochondrial dysfunction, underscoring its distinctive role in suppressing ferroptosis^42^. SLC7A11 is well established in inhibiting ferroptosis through importing cystine and promoting GSH biosynthesis^43^. The diverse regulatory mechanisms of SLC7A11 have been largely established in various types of cancer, including lung cancer, breast cancer and bladder cancer^44–47^. In lung cancer and bladder cancer, oncoproteins regulate tumor progression by promoting SLC7A11 transcription levels^45,47^. While in breast cancer, Metformin suppresses tumor growth through maintaining the protein stability of SLC7A11^46^. Therefore, SLC7A11 could promote tumor progression in multiple manners. In the present study, both PCBP1 and SLC7A11 are downstream molecules of ZDHHC9, which implies that there may be a close association between palmitoylation and ferroptosis. Study has shown that ZDHHC8-mediated SLC7A11 S-palmitoylation is critical for ferroptosis resistance during glioblastoma tumorigenesis^48^. In hepatocellular carcinoma, palmitoylation of SLC7A11 can modulate the antitumor efficacy of sorafenib^49^. Therefore, while current research on the link between palmitoylation and ferroptosis is limited, their interplay is universal across tumor types and may provide a potential strategy for tumor therapy.

This study has certain limitations. First, due to difficulties in clinical specimen acquisition, only 3 pairs of matched samples were used for label-free proteomic analysis. The inclusion of a larger cohort would likely enhance the statistical reliability of our findings. Second, although we have comprehensively delineated the ASF1B/ZDHHC9/PCBP1/SLC7A11 signaling axis that drives GCLM, the mechanistic investigation remains insufficient. For instance, we have attempted to identify the upstream molecules of ASF1B, but none have been found so far. Furthermore, we were unable to conduct studies on protein inhibitors, which prevents us from advancing translational research immediately. In future studies, we will strive to address these limitations to make our basic research more comprehensive and endowed with translational potential.

In summary, this study identified ASF1B as a critical target in GCLM and elucidated its regulatory mechanism. ASF1B promotes GCLM progression and inhibits ferroptosis via the ZDHHC9/PCBP1/SLC7A11 axis. Therapeutic strategies targeting this axis may enhance GCLM treatment efficacy and improve patient prognosis.

## Methods

### Patients, tissue microarray, and tissue samples

A total of 107 GC tissues and 22 randomly selected adjacent normal tissues were collected from the Department of General Surgery at the First Affiliated Hospital of Anhui Medical University between October 2012 and December 2013 for tissue microarray (TMA) analysis. This study was conducted with the approval of the Ethics Committee of the First Affiliated Hospital of Anhui Medical University. All GC and gastric cancer liver metastasis tissues were pathologically confirmed and staged according to the tumor lymph node metastasis (TNM) staging system, as outlined in the 7th edition of AJCC TNM staging system. Protein expression levels in the TMA were independently assessed by two pathologists who were blinded to the clinical information of the patients. This project has been approved by the Biomedical Ethics Committee of the First Affiliated Hospital of Anhui Medical University, with the biomedical ethics approval number 2023451. The relevant attachments have been uploaded along with the supplementary materials.

### Cell culture

The normal human gastric mucosal epithelial cell line-1 (GES-1), along with GC cell lines MKN28, HGC27, MKN45, AGS, and MGC803, were obtained from Genechem (Genechem, Shanghai, China). Cell cultures were maintained in RPMI-1640 medium (Corning, New York, USA) supplemented with 10% FBS (Clark, Frankfurt, Germany), penicillin, and streptomycin (HyClone, Utah, USA) in a humidified incubator at 37 °C with 5% CO_2_. All cell lines were tested to ensure they were free of mycoplasma contamination.

### Total RNA extraction and qPCR

Total RNA extraction was performed using TRIzol reagent (Thermo Fisher, Massachusetts, USA), and cDNA synthesis was carried out using the PrimeScript^TM^ RT Master Mix (Takara Bio Inc., Shiga, Japan). Quantitative PCR (QPCR) was conducted on a Roche detection system in a 20 µl reaction mixture containing SYBR Green. The relative mRNA expression levels were calculated using the 2 ^−ΔΔCt^ method, with GAPDH serving as the endogenous control. All primers used in this study were synthesized by Invitrogen. The specific primer sequences were as follows: PCBP1, F: 5′-GCCGGTGTGACTGAAAGTG-3′ and R: 5′-CCCAATGATGCTTCCTACTTC C-3′; GAPDH, F: 5′-ATCAAGAAGGTGGTGAAGCAGG-3′ and R: 5′-CGTCAAA GGTGGAGGAGTGG-3′.

### Cell lentivirus infection

All lentiviruses utilized in this study, including those for ASF1B and ZDHHC9 overexpression and ASF1B, ZDHHC9, and PCBP1 knockout (RNAi), were sourced from Genechem (Genechem, Shanghai, China). For lentiviral infection, MGC803, AGS, and HGC27 cells were seeded overnight in 6-well plates at a density of approximately 2 × 10^5^ cells per well. The following day, when cell confluence reached 30-50%, ASF1B and ZDHHC9 lentiviruses, along with negative controls (NC), were introduced into the wells. After two weeks of puromycin selection (2 mg/ml), stable cell lines for ASF1B-OE, shASF1B, ZDHHC9-OE, shZDHHC9, shPCBP1, and the NC group were established. Immunoblotting was performed 72 hours post-infection to assess the efficiency of overexpression or knockout. The shRNA sequences for ASF1B were as follows: shASF1B#1: 5′- CUGGAGUGG AAGAUCAUUUAU-3′, shASF1B#2: 5′-AGGGAGACACAUGUUUGUCUU -3′, shASF1B#3: 5′-GGUGACCCG CUUCCAUAUCAA-3′. The shRNA sequences for ZDHHC9 were: shZDHHC9#1: 5′- GAGGAACUACCGCUACU UCUA-3′, shZDHHC9#2: 5′- GAAGCAGCU UUCAUAGAAA-3′, shZDHHC9#3: 5′- GCUUCUUGGAGACAUUGAA-3′. The shRNA sequences for PCBP1 were: shPCBP1#1: 5′- GCAAGUUUGGAUGCAUCUA-3′, shPCBP1#2: 5′- GGAAAGGCGGGUGUAAGAU-3′, shPCBP1#3: 5′- GGAAG UAGGAAGCAUCAUU-3′.

### EdU assay

For the detection of 5-ethynyl-2′-deoxyuridine (EdU) incorporation, the BeyoClick EdU Cell Proliferation Kit (Cat# C0078S, Beyotime, Shanghai, China) was employed. Infected GC cells were first seeded onto slides within a 24-well plate and incubated overnight. On the following day, cells were treated with 10 µM EdU at 37 °C in a 5% CO2 atmosphere for 2 hours. The click reaction solution was then applied to the EdU-labeled cells, followed by a 30-minute incubation at room temperature. Finally, cells were stained with Hoechst 33342 (1:1000) for 10 minutes and subsequently observed and analyzed using a Leica microscope.

### Transwell assays

In the invasion assay, Matrigel was evenly spread on the upper surface of the membrane at the bottom of the Transwell chamber and allowed to polymerize into a gel by incubating at 37 °C for 4-5 hours. After removing the excess Matrigel, a serum-free cell suspension was prepared, and 100 µL was added to the Transwell chamber. The lower chamber was filled with 650 µL of culture medium containing 20% FBS, ensuring no bubbles formed during the process. Cells were cultured under standard conditions for 36-48 hours. Transwell chambers were then removed, and the cells were fixed with 4% paraformaldehyde for 30 minutes. The cells were stained with 0.1% crystal violet for 20 minutes, followed by the removal of non-migrating cells from the upper side of the membrane. Non-migrated cells were removed by mechanical means (the cotton swab method); specifically, a moistened cotton swab was used to wipe away the non-migrated cells with unidirectional rotation. After washing with PBS three times, the cells on the lower side of the membrane were counted under an inverted microscope. The migration assay followed the same protocol as the invasion assay, except without the use of Matrigel.

### Dual luciferase assay

In the dual luciferase assay, the Dual Luciferase Reporter Gene Assay Kit (Cat# 11402ES60, YEASEN, Shanghai, China)and the Hieff TransTM Liposomal Transfection Reagent (Cat# 40802ES01, YEASEN, Shanghai, China) were employed. Indicated GC cells were seeded into 96-well plates and cultured overnight. The following day, the constructed reporter gene plasmids were transfected into GC cells. Next day, 20 μL cell lysate was added into the black label plate, and then 100 μL firefly luciferase reaction solution and Renilla luciferase reaction solution were added into the lysate and detect the activity of luciferase, respectively.

### Acyl-biotin exchange (ABE) assay

For protein sample preparation, 1 × 10^7^ cells were lysed in 1 ml of lysis buffer (50 mM Tris-HCl pH 7.4, 5 mM EDTA, 150 mM NaCl, 2.5% SDS, protease inhibitor mixture). The lysate was sonicated using a probe sonicator, and the protein concentration of the supernatant was determined with the BCA protein assay kit. Each sample was treated with 10 mM TCEP for 30 min, followed by the addition of 25 mM n-ethylmaleimide (NEM). The sample was vortexed at room temperature for 2 h, then precipitated using chloroform/methanol/water (1:4:3, v/v/v), briefly air-dried, and dissolved in 1 ml of lysis buffer containing 5 mM biotin-HPDP. The sample was split into two equal parts and incubated with either 0.5 ml of 1 M hydroxylamine (HA) or a negative control (1 M NaCl) at room temperature for 3 h. The samples were centrifuged to precipitate, then dissolved in 200 µL of resuspension buffer (50 mM Tris-HCl pH 7.4, 2% SDS, 8 M urea, 5 mM EDTA). A control sample of 20 µL was set aside, and the remaining 180 µL was diluted 1:10 with 1 × PBS and incubated with 20 µL streptavidin beads at 4 °C with overnight shaking. The beads were washed three times with 1 × PBS containing 1% SDS, then mixed with SDS loading buffer and analyzed by western blot following a 10 min incubation in a 95 °C metal bath.

### Western blot analysis, reagents, and antibodies

Total protein was extracted using Mammalian Protein Extraction Reagent (M-PER™) supplemented with protease and phosphatase inhibitors (Cat# 78501, Thermo Fisher, Massachusetts, USA). Equal amounts of protein were separated by 8–12% SDS-PAGE and subsequently transferred to PVDF membranes (Millipore, Massachusetts, USA). Following a 1 h blocking step with 5% skim milk in TBST at room temperature, the membranes were incubated with primary antibodies targeting ASF1B (1:1000, Cat# 22258-1-AP, Proteintech, Chicago, USA), ZDHHC9 (1:1000, Cat# ER63246, HUABIO, Hangzhou, China), PCBP1 (1:750, Cat# R389370, ZENBIO, Chengdu, China), SLC7A11 (1:1000, Cat# T57046, Abmart, Shanghai, China), Flag (1:50000, Cat# 20543-1-AP, Proteintech, Chicago, USA), ubiquitin (1:2500, Cat# ET1609-21, HUABIO, Hangzhou, China), and GAPDH (1:5000, Cat# ab9485, Abcam, London, England). The protease and phosphatase inhibitors were sourced from APExBIO (APExBIO, Houston, USA), while PVDF membranes were obtained from Millipore (Millipore, Massachusetts, USA). Additional reagents included Dynabeads™ Protein G, Lipofectamine 2000, Tween-20, PBS, and TBS, all from Invitrogen (Invitrogen California, United States).

### Immunohistochemistry

Immunohistochemistry was conducted as previously described to quantify ASF1B, ZDHHC9, and SLC7A11 expression^50^. The staining intensity was graded from 0 (none) to 3 (strong), and the staining area from 0 (none) to 4 (76–100%). The overall staining score, calculated as the product of intensity and area, indicated high expression (≥5) or low expression (0-4) for ASF1B, ZDHHC9, and SLC7A11. Two pathologists, blinded to patient clinical information, independently evaluated protein expression levels in the TMAs.

### Coomassie blue staining

Coomassie blue staining was utilized to assess protein content following immunoprecipitation. The SDS-PAGE gel was heated to remove SDS, then incubated with BeyoBlue™ Coomassie Brilliant Blue Super Fast Staining Solution (Cat# P0017F, Beyotime, Shanghai, China) at room temperature for 30 min. Decolorization was performed with deionized water until the background of the SDS-PAGE gel was clear.

### Co-immunoprecipitation

Three transfected GC cell lines and corresponding control groups were prepared in advance and transferred to 100 mm culture dishes. Upon reaching 90–100% confluence, the cells were washed with PBS and lysed with pre-cooled M-PER protein lysis buffer at a concentration of 10^7^ cells/mL. Following centrifugation, the supernatant was collected and divided into three groups. One group served as the positive control (input group), while homologous IgG was added to the second group as a blank control. The supernatant from the third group was combined with ZDHHC9 monoclonal antibody and Protein G beads, forming an antigen-antibody complex using the immunoprecipitation kit. The protein mixture was incubated at 4 °C for 4–5 h on a rotary incubator. After incubation, a magnetic rack was used to precipitate the Protein G, primary antibody, and target protein complexes. Finally, the protein complexes from all three groups were denatured at 95 °C, and protein interactions were analyzed via Western blot.

### Flow cytometry

Flow cytometry (FCM) was performed following the manufacturer’s instructions. For cell cycle analysis, collected 5 × 10^5^ cells were fixed with 70% ethanol for 15 min and then washed with PBS. The cell pellet was resuspended in 500 µL PI solution (BD Biosciences, New Jersey, USA) and incubated at 37 °C for 40 min. After adding 3 mL PBS and washing, the cells were resuspended, and the supernatant was removed. The pellet was then resuspended in 500 µL PBS for FCM analysis. For apoptosis analysis, the PE Annexin V apoptosis detection kit (Beyotime, Shanghai, China) was used according to the manufacturer’s protocol. Briefly, after two washes with cold PBS, the cells were resuspended in a binding buffer. A total of 1 × 105 cells (100 µL) were transferred to a flow tube, and 5 µL PE Annexin V and 5 µL 7-AAD were added. The mixture was gently vortexed and incubated in the dark at room temperature for 15 min, followed by the addition of 400 µL binding buffer. The samples were then analyzed using a Cytoflex FCM (Beckman, USA).

### TUNEL assay

The TUNEL staining method was employed to assess tissue apoptosis levels in processed paraffin-embedded subcutaneous tumor tissues using a TUNEL kit (C1098, Beyotime, Shanghai, China). TUNEL staining was evaluated at 200 × magnification in six randomly selected areas using a Leica microscope.

### Protein ubiquitination detection

Transfected GC cells were seeded into 6-well plates and cultured overnight. The following day, the cells were treated with 5 μM Mg132 (Cat# HY-13259, MCE, New Jersey, USA) for 24 h. Immunoprecipitation was performed to enrich PCBP1 protein, and cell lysates were subsequently collected for Western blot analysis using an anti-ubiquitin antibody (Cat# 10201-2-AP, Proteintech, Chicago, USA).

### Cycloheximide (CHX) chase assay

CHX chase assay was performed to assess the stability of the SLC7A11 protein in PCBP1 knockdown cells. 1×10^6^ GC cells were pre-treated with rapamycin for 24 h and subsequently treated with 20 μg/mL of CHX (Cat# HY-12320, MCE, New Jersey, USA) for 0, 2, 4 and 8 h. Cell lysates were collected for western blotting.

### MDA and intracellular ROS detection

The concentration of MDA in GC cells was measured using the thiobarbituric acid method with the cell MDA assay kit (Cat#A003-4, Nanjing Institute of Bioengineering, Nanjing, China). MDA levels were calculated relative to cellular protein concentration and expressed as nmol MDA per milligram of protein (nmol/mg protein). Changes in intracellular ROS levels were assessed using the ROS assay kit (Cat#D6883, Sigma, Wisconsin, USA). 5×10^5^ GC cells cultured in 6-well plates were incubated with 5 μM DCFH-DA in serum-free medium at 37 °C for 30 min in the dark. After washing three times with PBS, the cells were resuspended in 500 μL PBS and analyzed via flow cytometry.

### Transmission electron microscopy (TEM)

The indicated cells were fixed in 4% paraformaldehyde (Cat#P0099, Beyotime, Shanghai, China) for 2 minutes. Following centrifugation, 2.5% glutaraldehyde (Cat#P1126, Solarbio, Beijing, China) was carefully added to the cell pellet along the tube wall. Ultra-thin sections were then prepared, and mitochondrial morphological changes were observed under transmission electron microscopy (TECNA I20, Philips, Eindhoven, Netherlands).

### Label-free proteomics detection

Three patients who underwent radical gastrectomy plus hepatectomy for GCLM during 2024 were selected for label-free proteomics analysis. For sample processing, dewaxed samples were scraped into centrifuge tubes, covered with BT lysis buffer, ammonium bicarbonate (ABC) solution, and PMSF, then subjected to non-contact ultrasonication at 4 °C for 10 min followed by brief centrifugation to collect the solution at the bottom. Trypsin and LysC were added for enzymatic digestion at 37 °C with shaking at 800 rpm, and desalted samples were analyzed post-digestion. For mass spectrometry, internal standards were prepared by mixing iRT (Biognosys, ThermoFisher) with samples at 1:20 (v/v); iRT, containing 11 synthetic peptides with optimized stability/retention time and no interference, was used for chromatographic calibration and quantitative QC. DIA raw data were processed using Spectronaut Pulsar with the uniprot-Homo sapiens-9606-2023.2.1 fasta database in library-free directDIA mode.

### RNA-sequencing

Following the construction of ASF1B knockdown and ZDHHC9 knockdown stable cell lines, both control and experimental group cells were seeded into 6-well plates. The next day, once the cells reached full confluence, 1 ml of TRIzol was added to each plate. A 1 ml pipette was used to thoroughly mix until a clear, non-viscous cell suspension was obtained. The cell suspension was then transferred into RNA-free cryovials for RNA-seq analysis.

### Immunoprecipitation tandem mass spectrometry (IP-MS)

HGC27 cells were first infected with a ZDHHC9 overexpression lentivirus. Immunoprecipitation was subsequently conducted as previously described, with the ZDHHC9-bound protein extracted using A/G PLUS agarose (Santa Cruz, Texas, USA). The protein sample was then washed using a buffer composed of 20 mM Tris HCl, 150 mM KCl, 1 mM dithiothreitol (DTT), 0.05% Nonidet P (NP) 40, 1 mM EDTA, 15% glycerol, and 0.2 mM PMSF. Finally, the samples were separated by SDS-PAGE and processed for MS analysis.

### Animal experimental methods

The animal experiments involved in this manuscript have been approved by the Animal Ethics Committee of the First Affiliated Hospital of Anhui Medical University, with the approval number: LLSC20231296. The relevant attachments have been uploaded along with the supplementary materials. Specifically, experimental BALB/c mice (6–8 weeks old, 18–22 g, half male/half female) were housed in an SPF facility (22 ± 2 °C, 50 ± 5% humidity, 12 h light/dark cycle) with ad libitum standard chow and sterile water, caged 3–5 per group (same sex) and acclimatized for 1 week; mice were assigned to 3 groups (n = 15/group) via stratified randomization (by weight, using Excel-generated random numbers) to avoid bias; single-blind (operators unaware of groups during tumor inoculation/drug administration) protocols were used to reduce observer bias; for surgery, mice were anesthetized with 2% isoflurane inhalation (monitored by reflex/respiratory rate), and euthanized via intraperitoneal injection of 150 mg/kg sodium pentobarbital (per GB/T 39766-2021) to ensure minimal pain. All these details are fully integrated into the revised manuscript with no critical information omitted.

### Tumor xenograft experiments

For xenograft model construction, six-week-old male BALB/c nude mice were obtained from the Shanghai Experimental Animal Center and randomly assigned to experimental and control groups (n = 6/group). 1×10^6^ transfected cells were subcutaneously injected into the lower left forelimb. Tumor size and volume were measured every 4 to 5 days to monitor growth and general health. At the designated endpoint, the mice were euthanized, and tumor growth curves were generated from the collected data. Tumor tissues were then fixed in 4% paraformaldehyde for further analysis, including hematoxylin and eosin (HE) staining, immunohistochemistry (IHC), and TUNEL staining.

### GC liver metastasis

In a separate procedure, 6-week-old BALB/c nude mice were prepared for splenic injection. The surgical area was disinfected with alcohol swabs, and an oblique incision approximately 1 cm below the left rib arch was made to expose the spleen. The distal end of the spleen was gently clamped to straighten it slightly. A 5 × 10^6^/20 µL cell suspension was injected into the middle of the spleen, ensuring the appearance of white spots along the needle’s path to confirm successful injection. If leakage occurred, the needle angle was promptly adjusted. After completing the injection, the needle was slowly withdrawn, and the injection site was gently pressed with saline-soaked gauze for 1–2 min until no further liquid seepage was observed. The peritoneum and skin were sutured with absorbable sutures, ensuring proper alignment of the skin. The surgical area was disinfected again with alcohol, and liver metastasis was assessed using live imaging after 6 weeks.

### GC peritoneum metastasis

To evaluate peritoneal metastasis, 5 × 10^6^ transfected GC cells were injected into the abdominal cavity of 6-week-old BALB/c nude mice. About 6 weeks later, in vivo fluorescence imaging was performed to monitor peritoneal metastasis. And then, mice were euthanized and peritoneal metastatic tumors were recorded and analyzed.

### GC lung metastasis

A 5 × 10^6^/20 µL cell suspension were injected into the tail vein of 6-week-old BALB/c nude mice to observe lung metastasis. After approximately 6 weeks, the number of lung metastases were recorded and analyzed after euthanizing mice.

### Public data and bioinformatics analysis

ASF1B expression information in GC and normal adjacent tissues were downloaded from UALCAN database (http://ualcan.path.uab.edu/analysis.html). Survival analysis of the GC patients with different ASF1B expression levels was performed using Kaplan-Meier Plotter (https://kmplot.com/analysis).

### Statistical analysis

All experiments were repeated at least three times. Data were presented as mean ± standard deviation. All the performed statistical analyses are described in each figure legend. Statistical p-values were obtained by application of the appropriate statistical tests using the GraphPad Prism 8. For all tests, p <0.05 was considered significant (ns: not significant, *p <0.05, **p <0.01, ***p <0.001).

## Supplementary information

Supplementary material

## Data Availability

Raw data have been deposited to Proteome Xchange database and National Center for Biotechnology Information (NCBI) under the BioProject number PXD069269 and PRJNA1338994. no custom code or scripts used.

## Code availability

Bioinformatics analysis data for this study were retrieved from UALCAN (http://ualcan.path.uab.edu/analysis.html) and analyzed via Kaplan-Meier Plotter (https://kmplot.com/analysis) using their built-in tools; all readers can directly access the original data through these databases, with no code was used in this study.

## References

[1] Bray, F. et al. Global cancer statistics 2022: GLOBOCAN estimates of incidence and mortality worldwide for 36 cancers in 185 countries. *CA-Cancer J. Clin.* **74**, 229–263 (2024). 38572751 10.3322/caac.21834

[2] Yu, D. et al. Fecal Streptococcus alteration is associated with gastric cancer occurrence and liver metastasis. *mBio* **12**, e0299421 (2021). 34872346 10.1128/mBio.02994-21 PMC8649758

[3] Huang, C. et al. Laparoscopic vs open distal gastrectomy for locally advanced gastric cancer: five-year outcomes from the CLASS-01 randomized clinical trial. *JAMA Surg.* **157**, 9–17 (2022). 34668963 10.1001/jamasurg.2021.5104 PMC8529527

[4] Das, K. et al. Genomic predictors of chemotherapy efficacy in advanced or recurrent gastric cancer in the GC0301/TOP002 phase III clinical trial. *Cancer Lett.* **412**, 208–215 (2018). 29061504 10.1016/j.canlet.2017.10.011

[5] Fujitani, K. et al. Gastrectomy plus chemotherapy versus chemotherapy alone for advanced gastric cancer with a single non-curable factor (REGATTA): a phase 3, randomised controlled trial. *Lancet Oncol.* **17**, 309–318 (2016). 26822397 10.1016/S1470-2045(15)00553-7

[6] Li, H. et al. Utility of At-trastuzumab for the treatment of metastatic gastric cancer in the liver: evaluation of a preclinical α-radioimmunotherapy approach in a clinically relevant mouse model. *J. Nucl. Med.* **62**, 1468–1474 (2021). 33547212 10.2967/jnumed.120.249300 PMC8724896

[7] Wang, Q. et al. METTL3-mediated mA modification of HDGF mRNA promotes gastric cancer progression and has prognostic significance. *Gut* **69**, 1193–1205 (2020). 31582403 10.1136/gutjnl-2019-319639

[8] Li, D. et al. CST1 inhibits ferroptosis and promotes gastric cancer metastasis by regulating GPX4 protein stability via OTUB1. *Oncogene* **42**, 83–98 (2023). 36369321 10.1038/s41388-022-02537-x PMC9816059

[9] Wei, F. et al. SZDHHC7-mediated palmitoylation of ATG16L1 facilitates LC3 lipidation and autophagosome formation. *Autophagy* **20**, 2719–2737 (2024). 10.1080/15548627.2024.2386915 PMC11587844 39087410

[10] Tran, C. et al. Phosphatidylinositol 4-kinase III α governs cytoskeletal organization for invasiveness of liver cancer cells. *Gastroenterology* **167**, 522–537 (2024). 38636680 10.1053/j.gastro.2024.04.009

[11] Binhui, Z. et al. Protein palmitoylation in cancer: molecular functions and therapeutic potential. *Mol. Oncol.* **17**, 1 (2022). 10.1002/1878-0261.13308 PMC9812842 36018061

[12] Du, G. et al. ROS-dependent S-palmitoylation activates cleaved and intact gasdermin D. *Nature* **630**, 437–446 (2024). 38599239 10.1038/s41586-024-07373-5 PMC11283288

[13] Pei, X. et al. Palmitoylation of MDH2 by ZDHHC18 activates mitochondrial respiration and accelerates ovarian cancer growth. *Sci. China Life Sci.* **65**, 2017–2030 (2022). 35366151 10.1007/s11427-021-2048-2

[14] Zhang, Z. et al. DHHC9-mediated GLUT1 S-palmitoylation promotes glioblastoma glycolysis and tumorigenesis. *Nat. Commun.* **12**, 5872 (2021). 34620861 10.1038/s41467-021-26180-4 PMC8497546

[15] Le, X. et al. DNA methylation downregulated ZDHHC1 suppresses tumor growth by altering cellular metabolism and inducing oxidative/ER stress-mediated apoptosis and pyroptosis. *Theranostics* **10**, 9495–9511 (2020). 32863941 10.7150/thno.45631 PMC7449911

[16] Lin, Z. et al. Palmitoyl acyltransferase ZDHHC7 inhibits androgen receptor and suppresses prostate cancer. *Oncogene* **42**, 2126–2138 (2023). 37198397 10.1038/s41388-023-02718-2

[17] Liu, F. et al. Ubiquitination and deubiquitination in cancer: from mechanisms to novel therapeutic approaches. *Mol. Cancer* **23**, 148 (2024). 39048965 10.1186/s12943-024-02046-3 PMC11270804

[18] Jiang, H. et al. Protein semisynthesis reveals plasticity in HECT E3 ubiquitin ligase mechanisms. *Nat. Chem.* **16**, 1894–1905 (2024). 10.1038/s41557-024-01576-z PMC12087361 39030419

[19] Zheng, S. et al. Extracellular vesicle-packaged PIAT from cancer-associated fibroblasts drives neural remodeling by mediating m5C modification in pancreatic cancer mouse models. *Sci. Transl. Med.* **16**, eadi0178 (2024). 39018369 10.1126/scitranslmed.adi0178

[20] Zhou, X. et al. Bismuth sulfide nanoflowers facilitated miR339 delivery to overcome stemness and radioresistance through ubiquitin-specific peptidase 8 in esophageal cancer. *ACS Nano* **18**, 19232–19246 (2024). 38996055 10.1021/acsnano.4c05100

[21] Brunet, M. et al. The E3 ubiquitin ligase TRIP12 is required for pancreatic acinar cell plasticity and pancreatic carcinogenesis. *J. Pathol.* **263**, 466–481 (2024). 38924548 10.1002/path.6298

[22] Hong, J. et al. The PRMT6/STAT1/ACSL1 axis promotes ferroptosis in diabetic nephropathy. *Cell Death Differ.* **31**, 1–10 (2024). 39134684 10.1038/s41418-024-01357-8 PMC11519485

[23] Mo, C. et al. Dopaminylation of endothelial TPI1 suppresses ferroptotic angiocrine signals to promote lung regeneration over fibrosis. *Cell Metab.* **36**, 1839–1857.e1812 (2024). 39111287 10.1016/j.cmet.2024.07.008

[24] Cheng, X. et al. Atlas of metastatic gastric cancer links ferroptosis to disease progression and immunotherapy response. *Gastroenterology* **167**, 1–10 (2024). 39097198 10.1053/j.gastro.2024.07.038

[25] Schwab, A. et al. Zeb1 mediates EMT/plasticity-associated ferroptosis sensitivity in cancer cells by regulating lipogenic enzyme expression and phospholipid composition. *Nat. Cell Biol.* **26**, 1–11 (2024). 39009641 10.1038/s41556-024-01464-1 PMC11392809

[26] Zhang, L. et al. The EGR1/miR-139/NRF2 axis orchestrates radiosensitivity of non-small-cell lung cancer via ferroptosis. *Cancer Lett.* **595**, 217000 (2024). 38821254 10.1016/j.canlet.2024.217000

[27] Yan, H. et al. Targeting ferroptosis to treat colorectal cancer. *Trends Cell Biol.* **33**, 185–188 (2023). 36473802 10.1016/j.tcb.2022.11.003

[28] Zhao, X. et al. Apigenin-7-glucoside-loaded nanoparticle alleviates intestinal ischemia-reperfusion by ATF3/SLC7A11-mediated ferroptosis. *J. Control. Release* **366**, 182–193 (2024). 38145659 10.1016/j.jconrel.2023.12.038

[29] Cao, N. et al. LPCAT2 inhibits colorectal cancer progression via the PRMT1/SLC7A11 axis. *Oncogene* **43**, 1714–1725 (2024). 38605214 10.1038/s41388-024-02996-4 PMC11136653

[30] Du, Y. et al. APE1 inhibition enhances ferroptotic cell death and contributes to hepatocellular carcinoma therapy. *Cell Death Differ.* **31**, 431–446 (2024). 38418695 10.1038/s41418-024-01270-0 PMC11043431

[31] Sardana, S. et al. S-palmitoylation during retinoic acid-induced neuronal differentiation of SH-SY5Y neuroblastoma cells. *J. Proteome Res.* **22**, 2421–2435 (2023). 37294931 10.1021/acs.jproteome.3c00151 PMC10337253

[32] Yao, H. et al. Inhibiting PD-L1 palmitoylation enhances T-cell immune responses against tumours. *Nat. Biomed. Eng.* **3**, 1–10 (2019). 30952982 10.1038/s41551-019-0375-6

[33] Chen, C. et al. NAT10 promotes gastric cancer liver metastasis by modulation of M2 macrophage polarization and metastatic tumor cell hepatic adhesion. *Adv. Sci.* **12**, e2410263 (2025). 10.1002/advs.202410263 PMC12005778 39985269

[34] Tang, X. et al. Single-cell profiling reveals altered immune landscape and impaired NK cell function in gastric cancer liver metastasis. *Oncogene* **43**, 2635–2646 (2024). 39060439 10.1038/s41388-024-03114-0

[35] Zhao, Z. et al. Activation of the FOXM1/ASF1B/PRDX3 axis confers hyperproliferative and antioxidative stress reactivity to gastric cancer. *Cancer Lett.* **589**, 216796 (2024). 38537775 10.1016/j.canlet.2024.216796

[36] Jiangqiao, Z. et al. Anti-silencing function 1B histone chaperone promotes cell proliferation and migration via activation of the AKT pathway in clear cell renal cell carcinoma. *Biochem. Biophys. Res. Commun.* **511**, 165–172 (2019). 30777326 10.1016/j.bbrc.2019.02.060

[37] Zhang, W. et al. ASF1B promotes oncogenesis in lung adenocarcinoma and other cancer types. *Front. Oncol.* **11**, 731547 (2021). 34568067 10.3389/fonc.2021.731547 PMC8459715

[38] Jeong, D. W. et al. Palmitoylation-driven PHF2 ubiquitination remodels lipid metabolism through the SREBP1c axis in hepatocellular carcinoma. *Nat. Commun.* **14**, 6370 (2023). 37828054 10.1038/s41467-023-42170-0 PMC10570296

[39] Dai, X. et al. Post-translational regulations of PD-L1 and PD-1: mechanisms and opportunities for combined immunotherapy. *Semin. Cancer Biol.* **85**, 246–252 (2022). 33831533 10.1016/j.semcancer.2021.04.002 PMC8490479

[40] Jiang, L. et al. Elaborate cooperation of poly(rC)-binding proteins 1/2 and glutathione in ferroptosis induced by plasma-activated Ringer’s lactate. *Free Radic. Biol. Med.* **214**, 28–41 (2024). 38325565 10.1016/j.freeradbiomed.2024.02.001

[41] Luo, Y. et al. PCBP1 protects bladder cancer cells from mitochondria injury and ferroptosis by inducing LACTB mRNA degradation. *Mol. Carcinog.* **62**, 907–919 (2023). 37157950 10.1002/mc.23533

[42] Lee, J. et al. Poly(rC)-binding protein 1 represses erritinophagy-mediated ferroptosis in head and neck cancer. *Redox Biol.* **51**, 102276 (2022). 35290903 10.1016/j.redox.2022.102276 PMC8921323

[43] Koppula, P. et al. Cystine transporter SLC7A11/xCT in cancer: ferroptosis, nutrient dependency, and cancer therapy. *Protein Cell* **12**, 599–620 (2021). 33000412 10.1007/s13238-020-00789-5 PMC8310547

[44] Liu, J. et al. Deubiquitylase USP52 promotes bladder cancer progression by modulating ferroptosis through stabilizing SLC7A11/xCT. *Adv. Sci.* **12**, e2410263 (2024). 10.1002/advs.202403995 PMC11615784 39392373

[45] Zhang, W. et al. RBMS1 regulates lung cancer ferroptosis through translational control of SLC7A11. *J. Clin. Invest.* **131**, e152067 (2021). 34609966 10.1172/JCI152067 PMC8592553

[46] Yang, J. et al. Metformin induces ferroptosis by inhibiting UFMylation of SLC7A11 in breast cancer. *J. Exp. Clin. Cancer Res.* **40**, 206 (2021). 34162423 10.1186/s13046-021-02012-7 PMC8223374

[47] Shen, L. et al. PHGDH inhibits ferroptosis and promotes malignant progression by upregulating SLC7A11 in bladder cancer. *Int. J. Biol. Sci.* **18**, 5459–5474 (2022). 36147463 10.7150/ijbs.74546 PMC9461664

[48] Wang, Z. et al. AMPKα1-mediated ZDHHC8 phosphorylation promotes the palmitoylation of SLC7A11 to facilitate ferroptosis resistance in glioblastoma. *Cancer Lett.* **584**, 216619 (2024). 38211651 10.1016/j.canlet.2024.216619

[49] Shi, Z. et al. Loss of LncRNA DUXAP8 synergistically enhanced sorafenib-induced ferroptosis in hepatocellular carcinoma via SLC7A11 de-palmitoylation. *Clin. Transl. Med.* **13**, e1300 (2023). 37337470 10.1002/ctm2.1300 PMC10280000

[50] Lee, K. et al. ASF1a promotes non-homologous end joining repair by facilitating phosphorylation of MDC1 by ATM at double-strand breaks. *Mol. Cell* **68**, 61–75 (2017). 28943310 10.1016/j.molcel.2017.08.021 PMC5743198

