# Supplementary material for: ASF1B promotes gastric cancer liver metastasis through inhibiting ZDHHC9/PCBP1/ SLC7A11 signaling axis mediated ferroptosis
Source: NPJ Precis Oncol. 2026 Jan 14;10:66. doi: 10.1038/s41698-026-01272-w (PMC12905238; doi:10.1038/s41698-026-01272-w)
Supplement: Supplementary file 1 — Supplementary material [file 41698_2026_1272_MOESM1_ESM.pdf]

Supplementary Figures

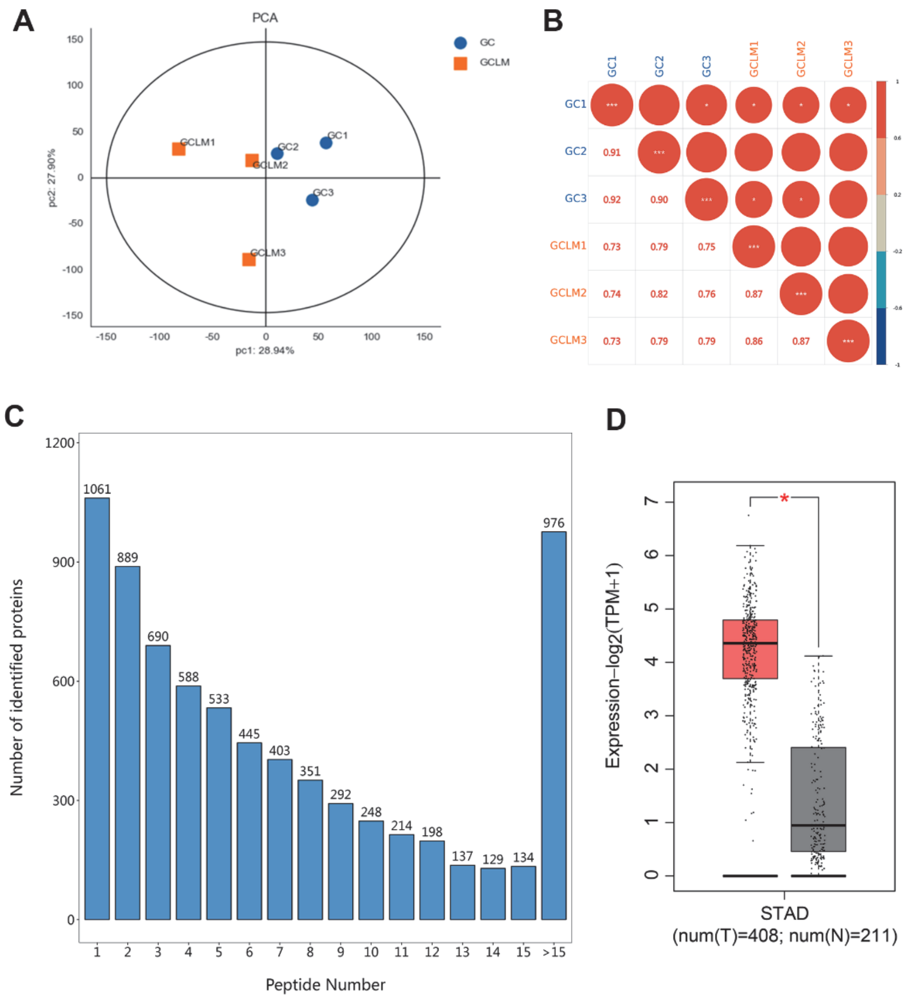

Supplementary Fig. S1 Sample stability validation for label-free proteomics analysis. A: Principal Component Analysis (PCA) analysis showed that a large amount of raw data was retained among the three pairs of GCLM samples, reducing inter group differences. B: Correlation analysis shows that there is little difference between samples with different GCLM. C: Analysis of the number of protein peptide segments in GCLM tissue samples.D: Ualcan database showed that ASF1B were upregulated in GC compared to normal tissue.

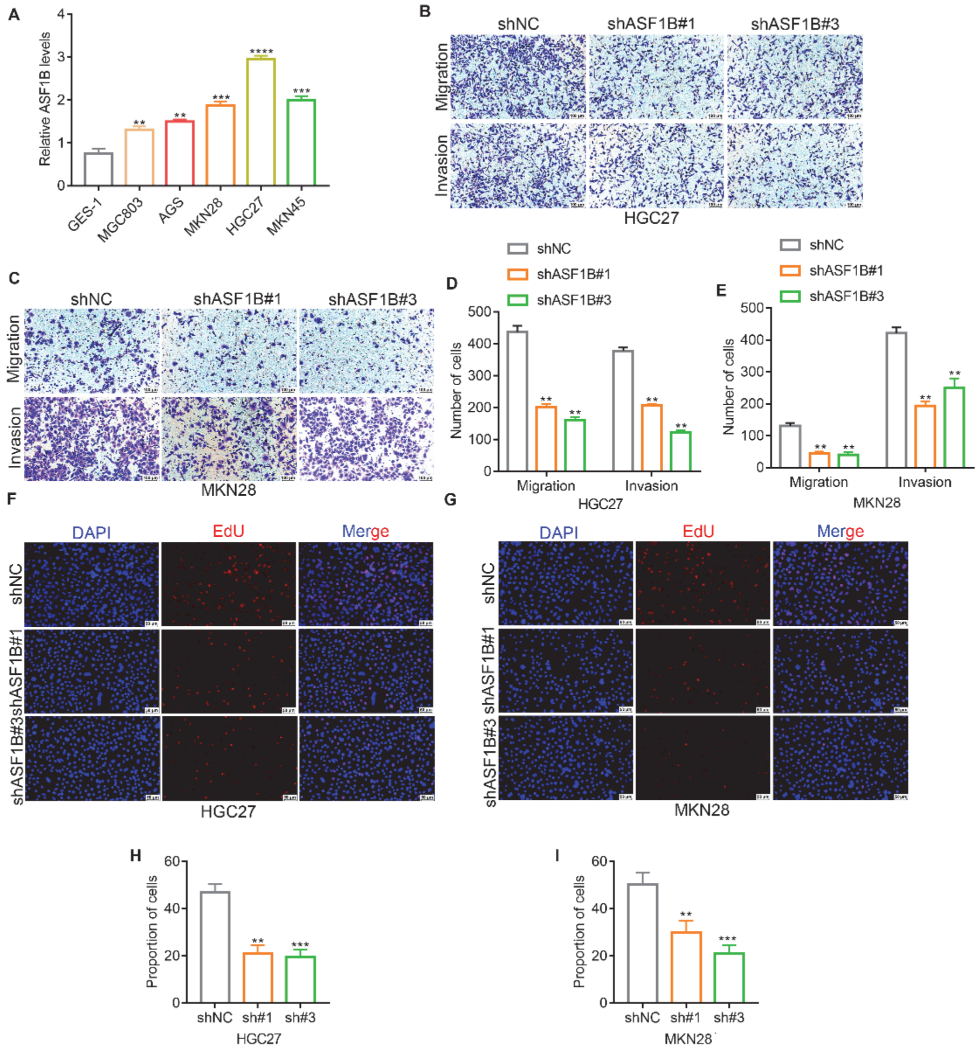

Supplementary Fig. S2 Overexpression of ASF1B promotes migration, invasion, and proliferation of GC cells. A:ASF1B protein expression was significantly up-regulated in GC cell lines. B-E: Transwell assay showed that knocking out ASF1B significantly reduced the migration and invasion of MKN28 and HGC27. F-I: EdU analysis demonstrated that knocking out ASF1B significantly reduced the cell proliferation ability of MKN28 and HGC27.

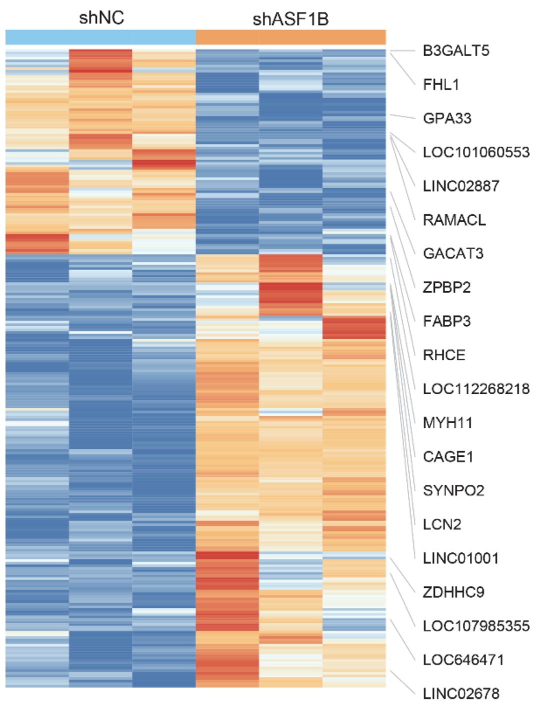

Supplementary Fig. S3 Heat map shows a correlation between ASF1B and ZDHHC9 expression.

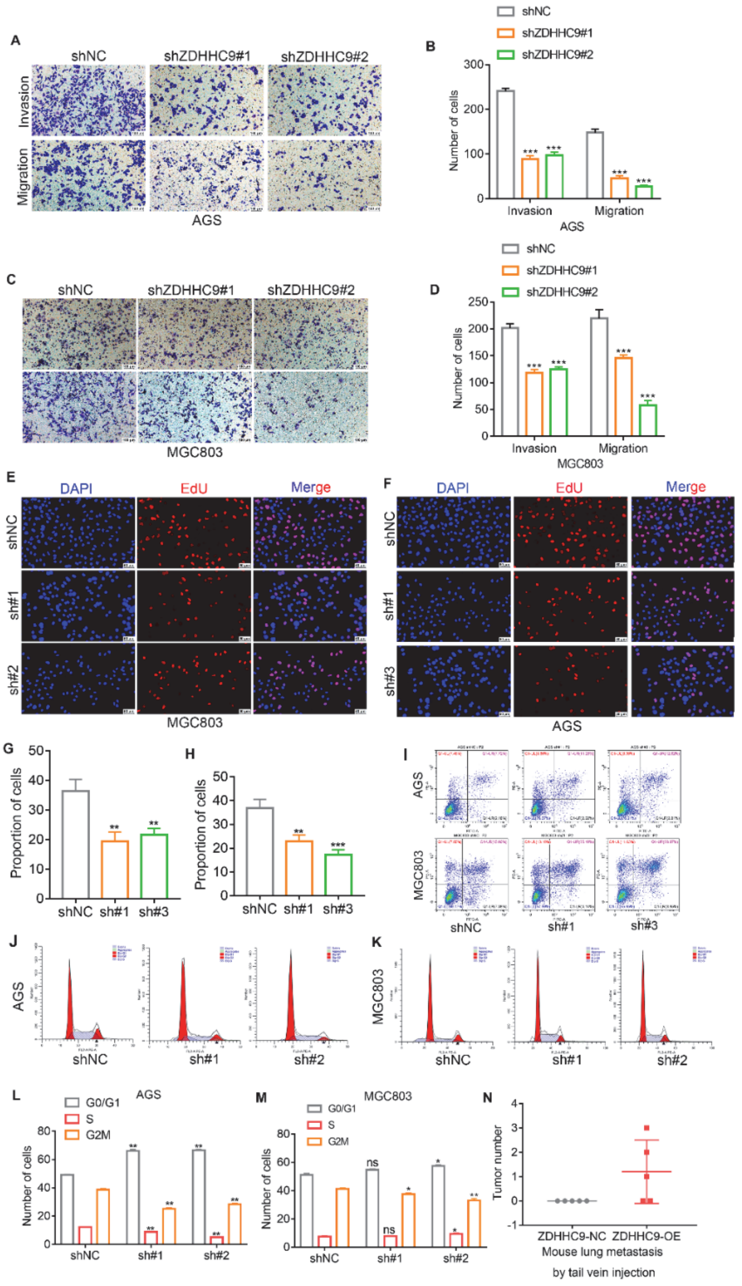

Supplementary Fig. S4 Overexpression of ZDHHC9 promotes malignant phenotype of GC cells and promotes tumor metastasis to the liver and lung. A-D: Transwell assays indicated that overexpression of ZDHHC9 significantly enhances the migration and invasion characteristics of HGC27 cells, and it was inhibited after silencing ZDHHC9. E-H: EDU results showed that overexpression of ZDHHC9 increased cell proliferation, while silencing ZDHHC9 inhibited cell proliferation. I: Silencing ZDHHC9 increases GC cell apoptosis. J-M: Silencing ZDHHC9 inhibits cell cycle progression. N: Overexpression of ZDHHC9 increases the number of lung metastases.

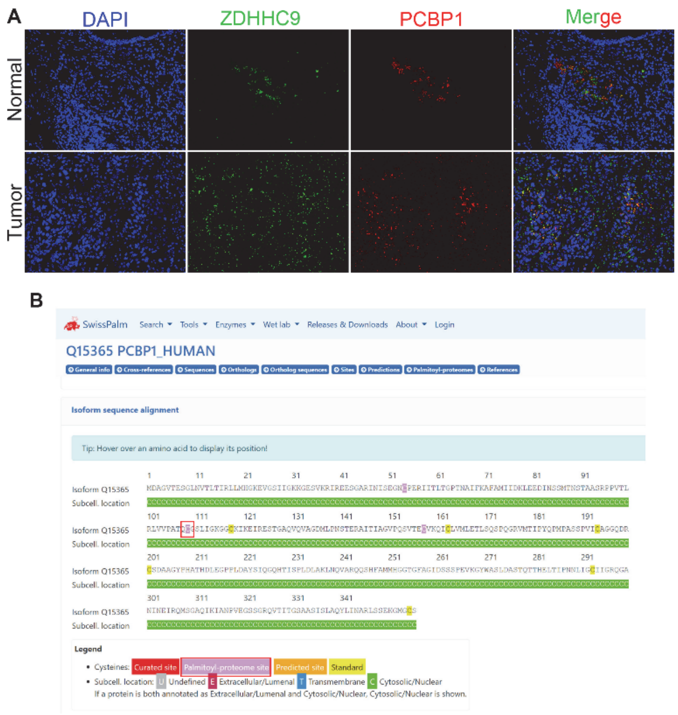

Supplementary Fig. S5: A: Immunofluorescence also showed that PCBP1 and ZDHHC9 are co-localized in GC tissues (Supplementary Fig. S5A). B: Using the Swisspalm palmitoylation prediction tool (https://swisspalm. org), three potential palmitoylation sites in PCBP1 were identified: Cys109, Cys54, and Cys158.

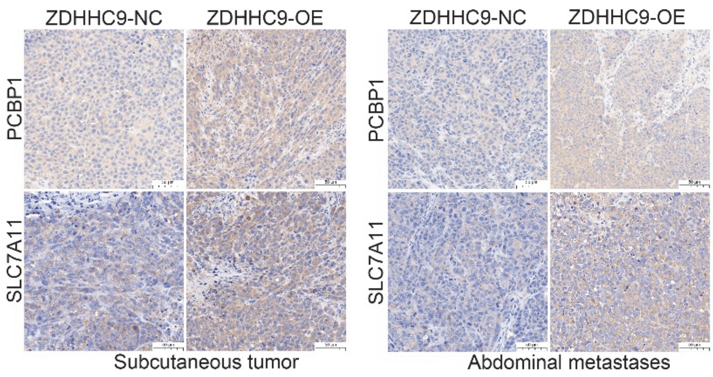

Supplementary Fig. S6: IHC reconfirmed that ZDHHC9 regulated the expression of SLC7A11 and PCBP1 in vivo.

Figure2A

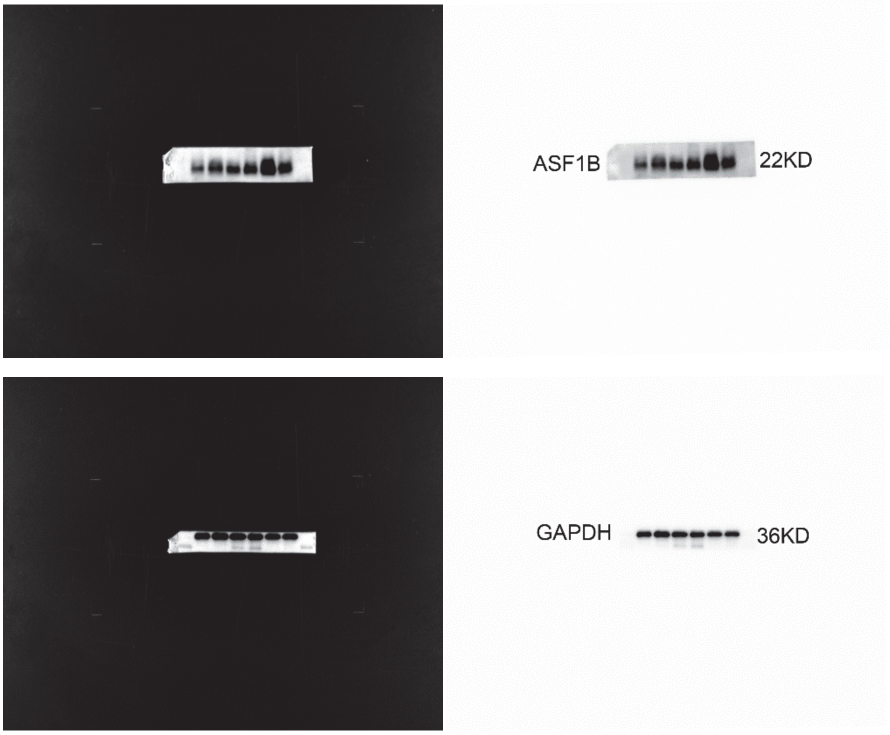

Figure2B

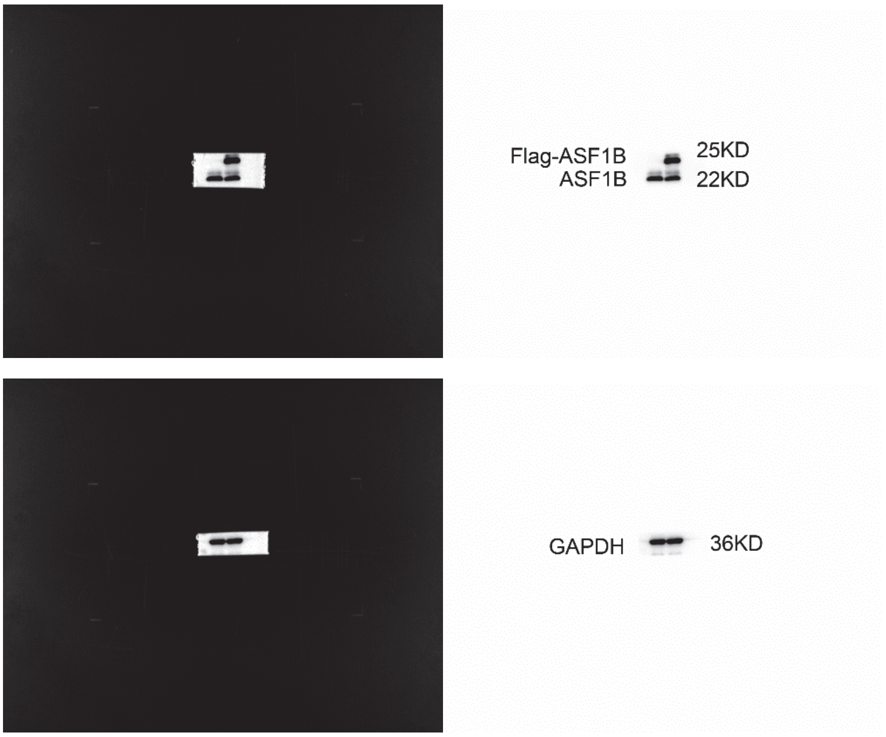

Figure2C

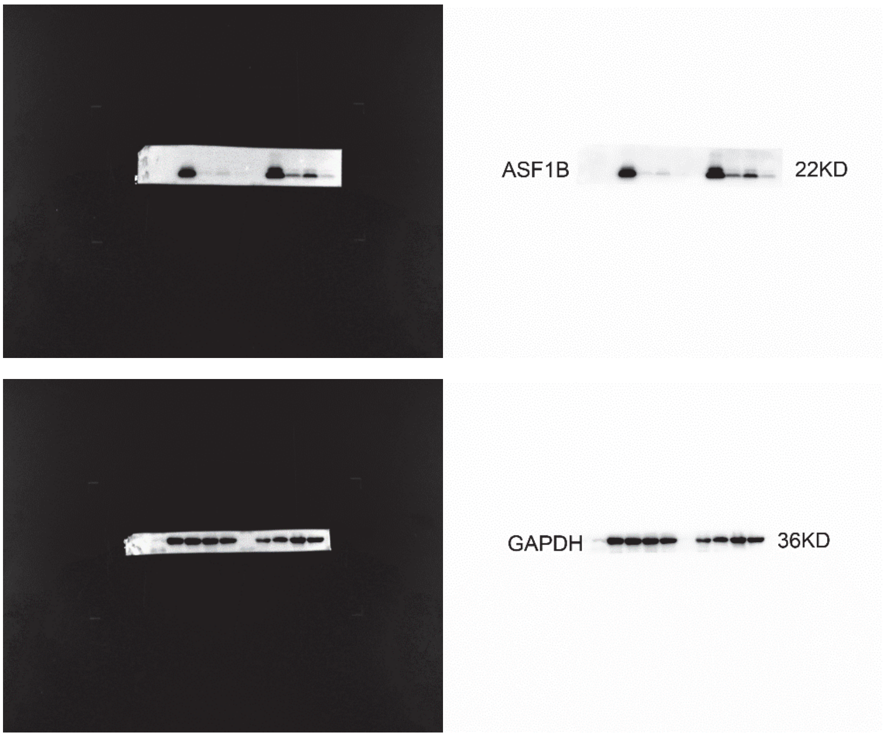

Figure3J

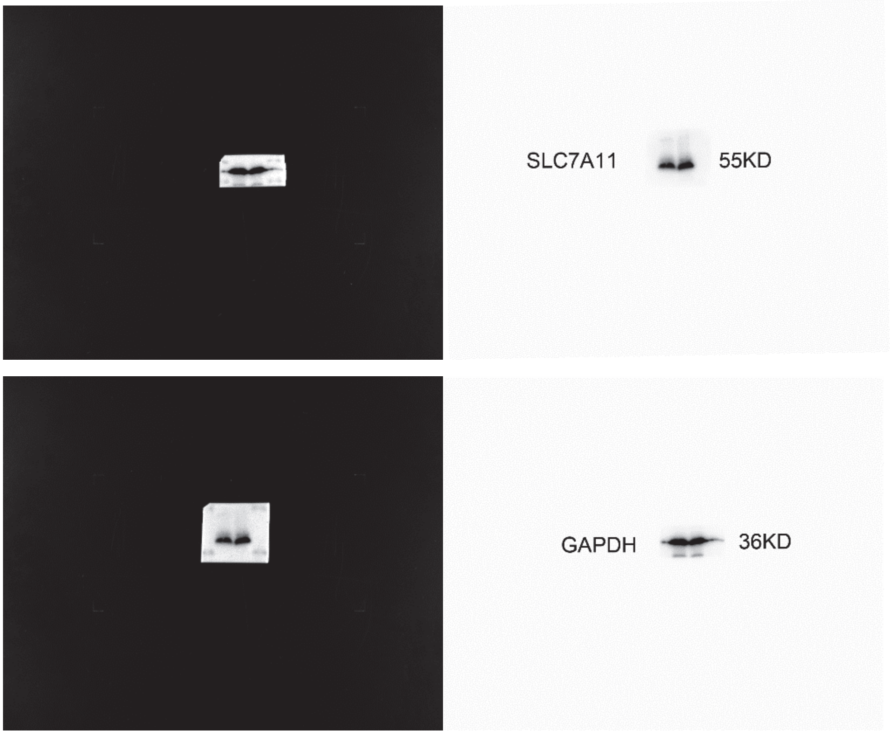

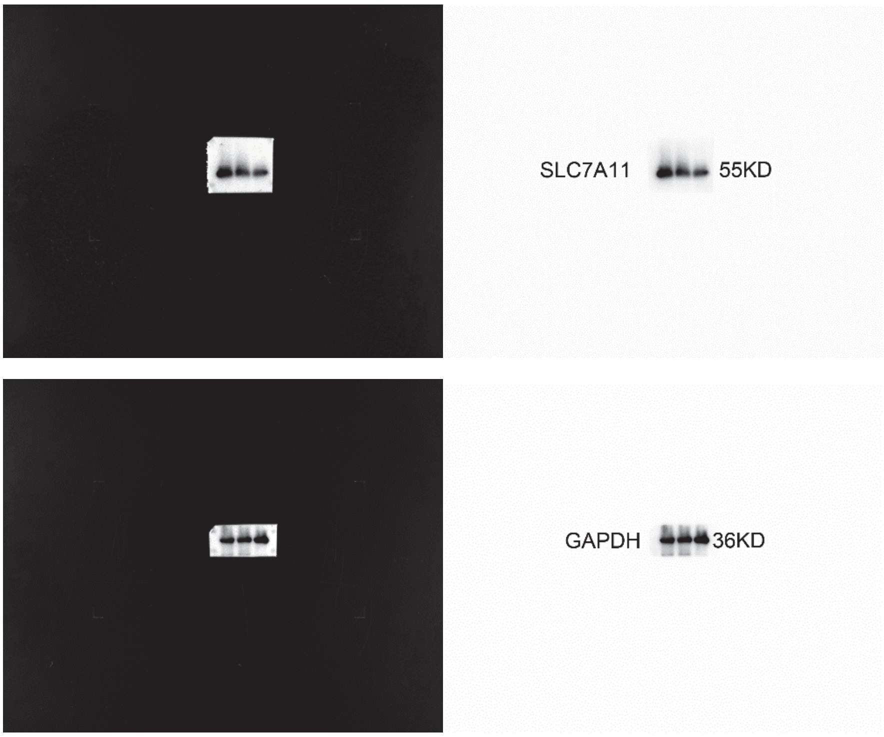

Figure4D

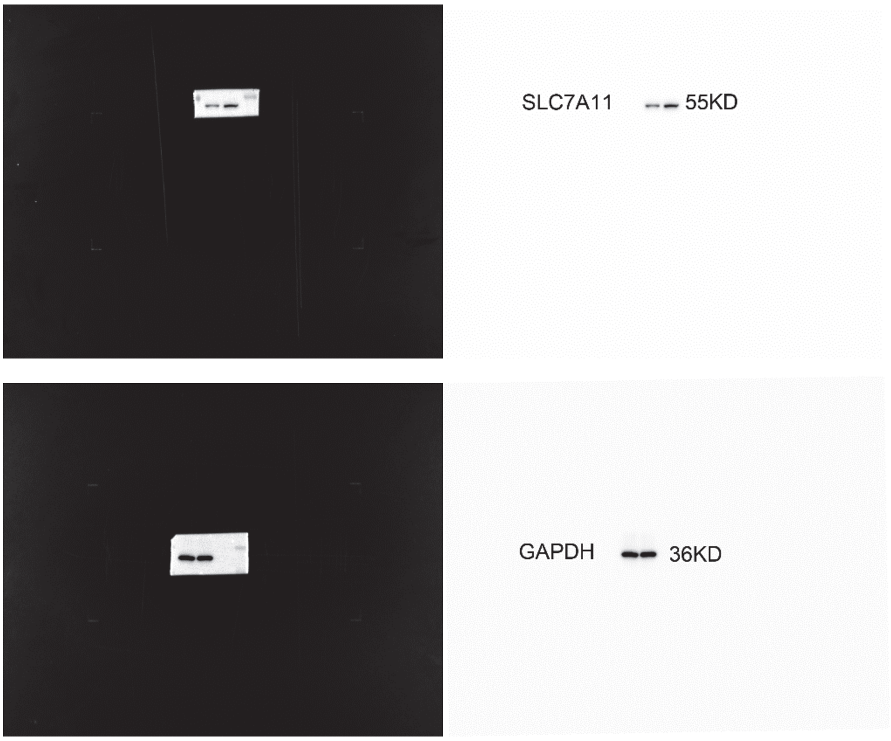

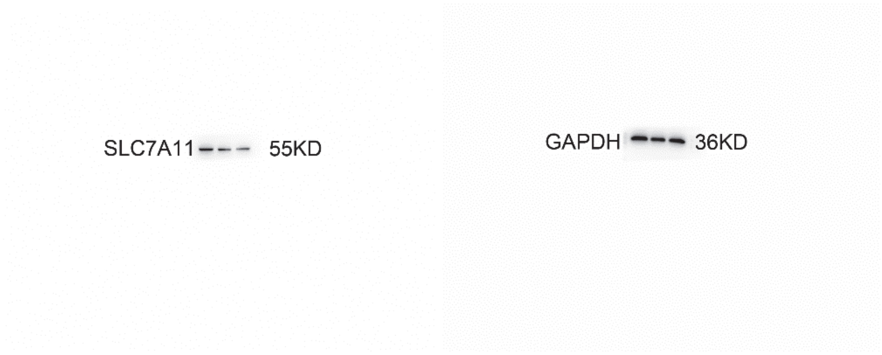

Figure4G

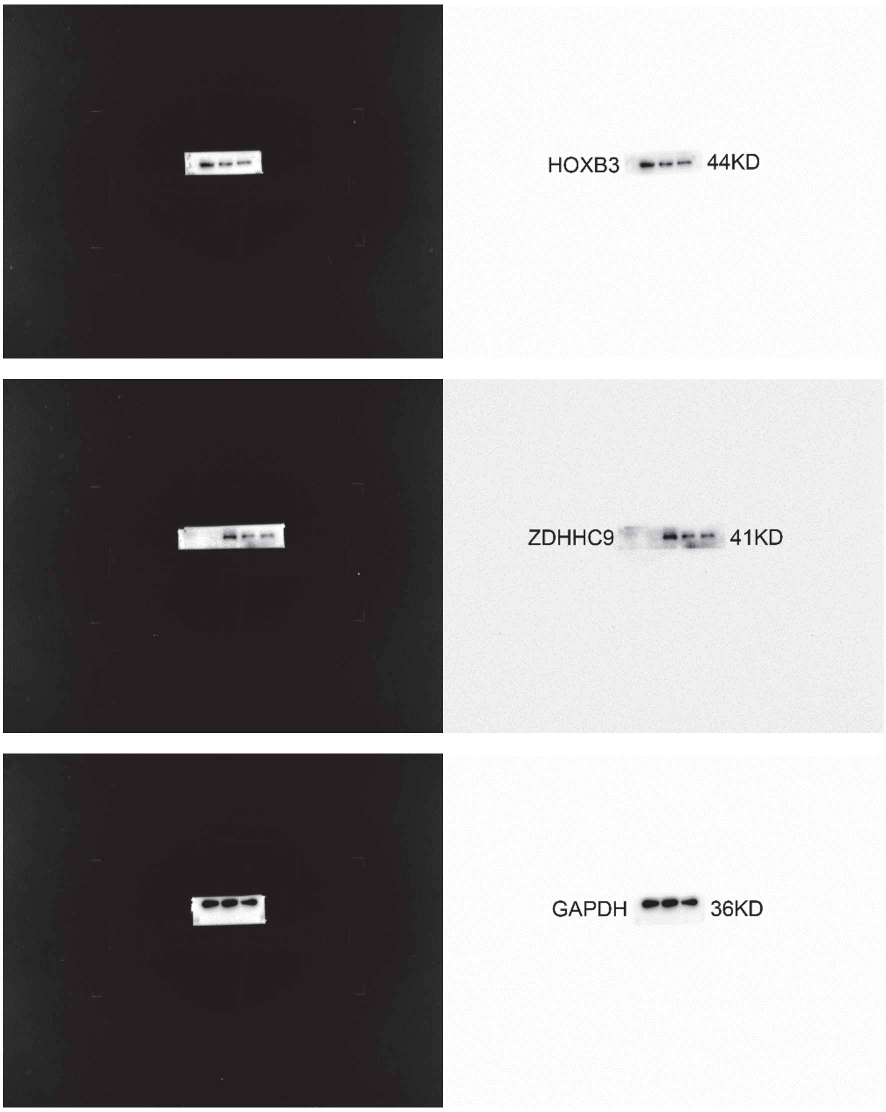

Figure4H

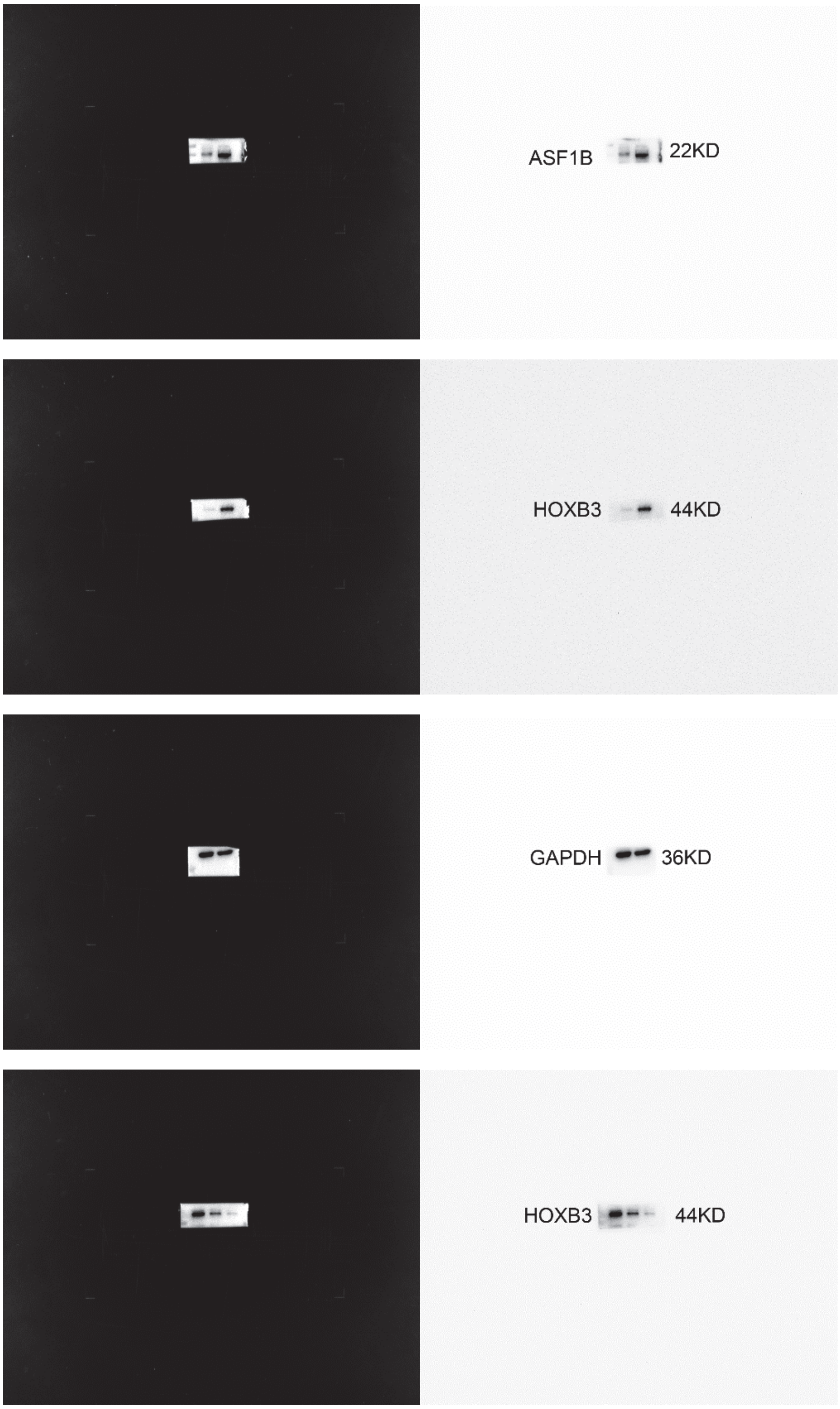
ASF1B
22KD
HOXB3
44KD
GAPDH
36KD
HOXB3
44KD

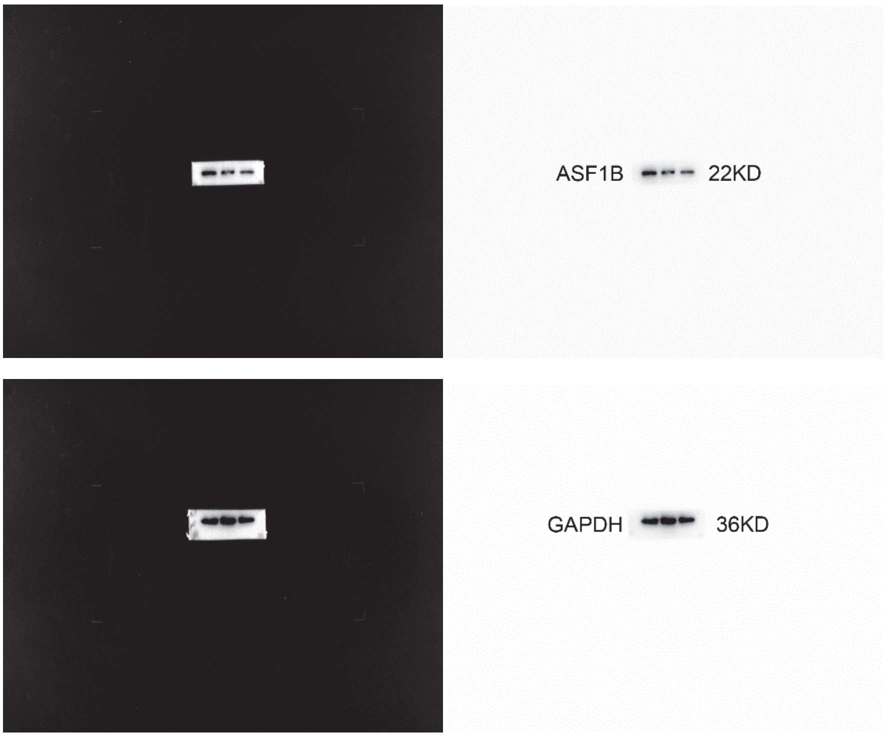

Figure4J

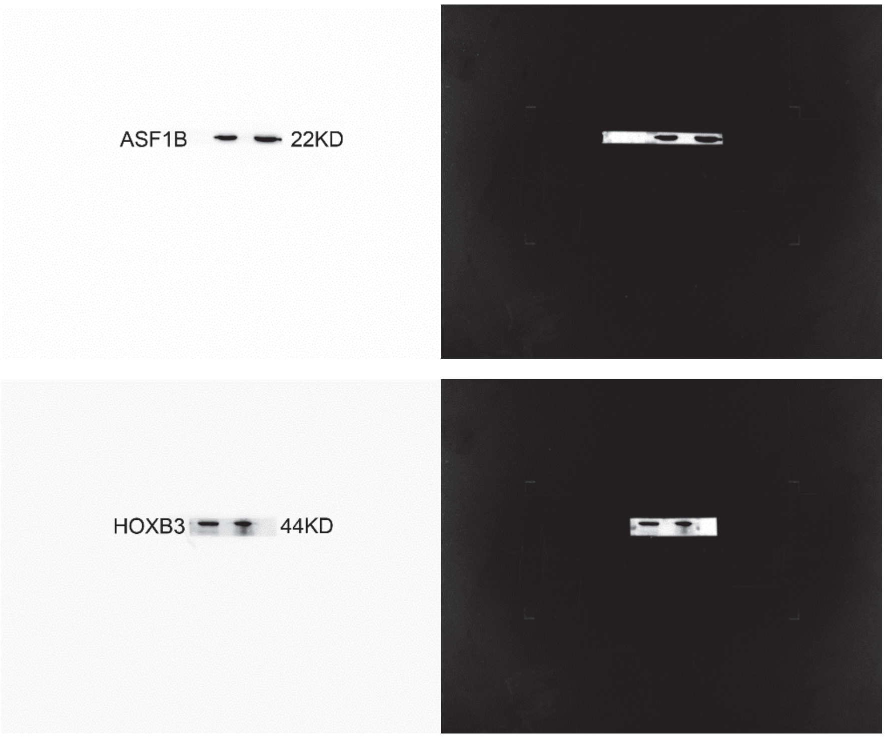

Figure6C

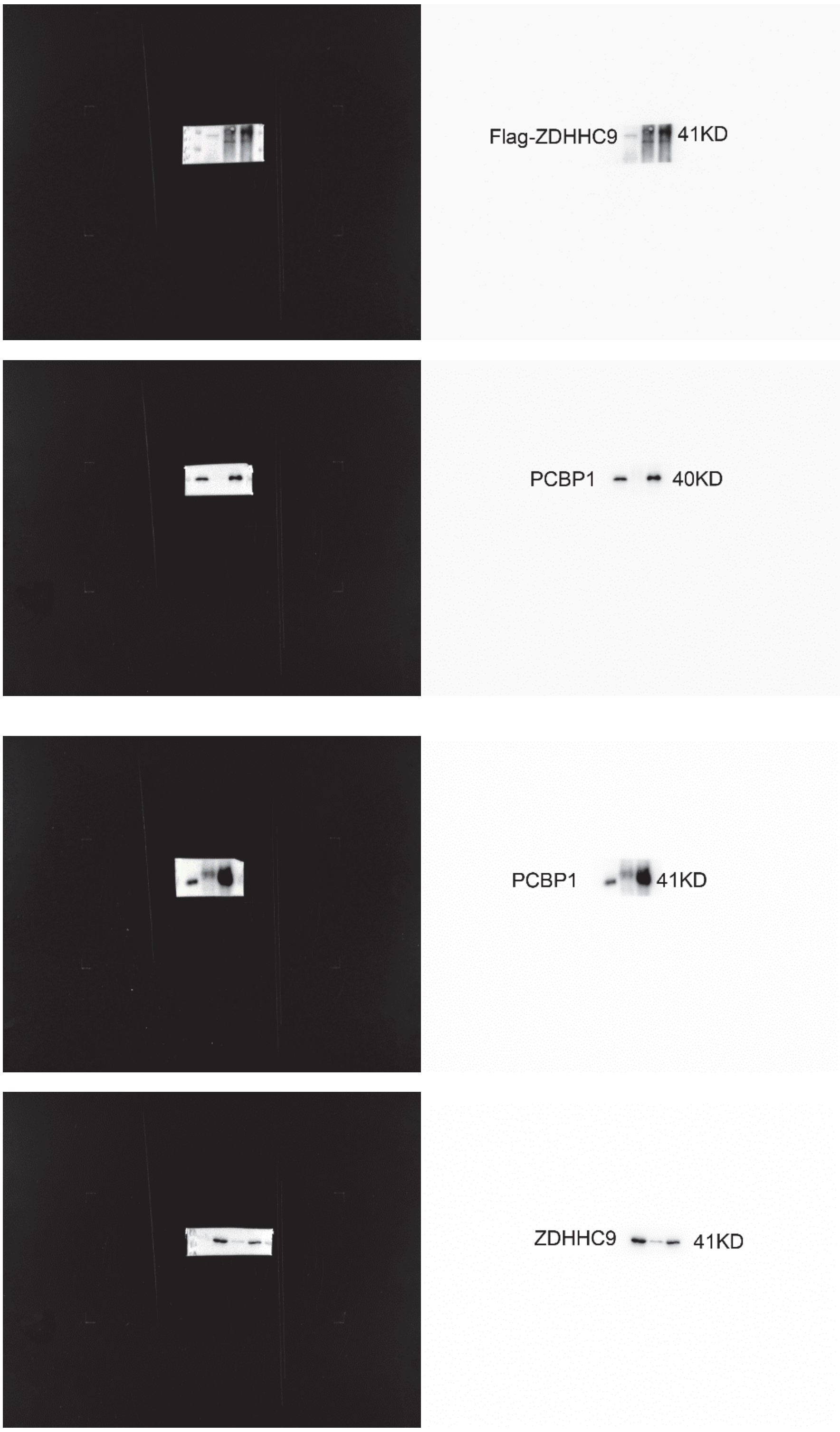

Figure6G

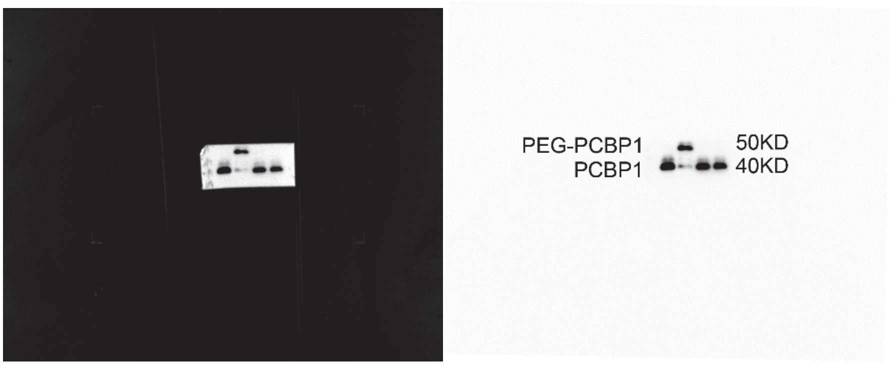
PEG-PCBP1
PCBP1
50KD
40KD

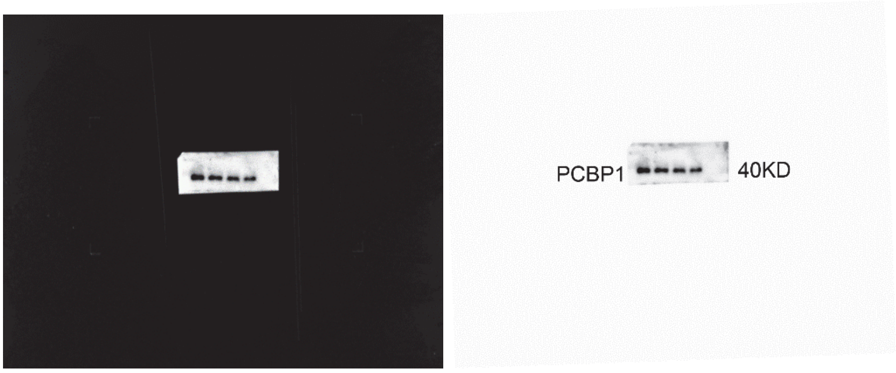
PCBP1
40KD

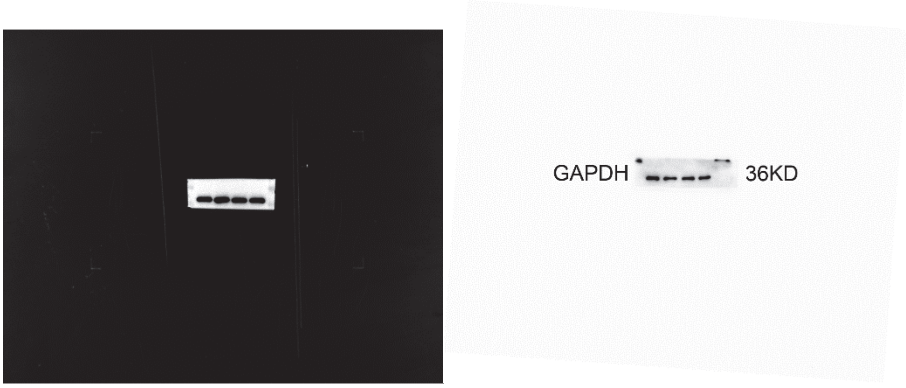
GAPDH
36KD

Figure6H

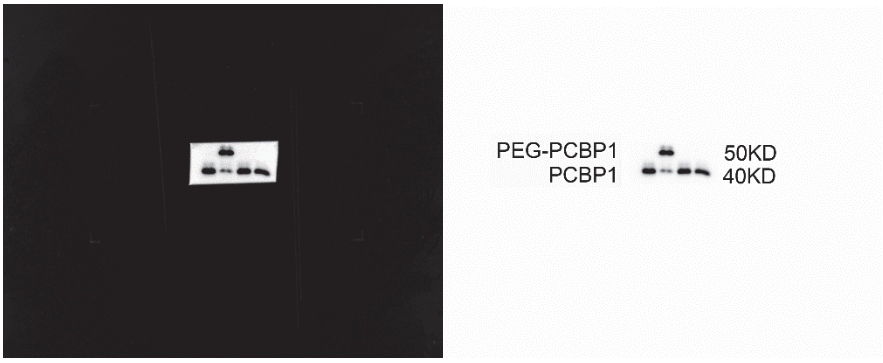
PEG-PCBP1
PCBP1
50KD
40KD

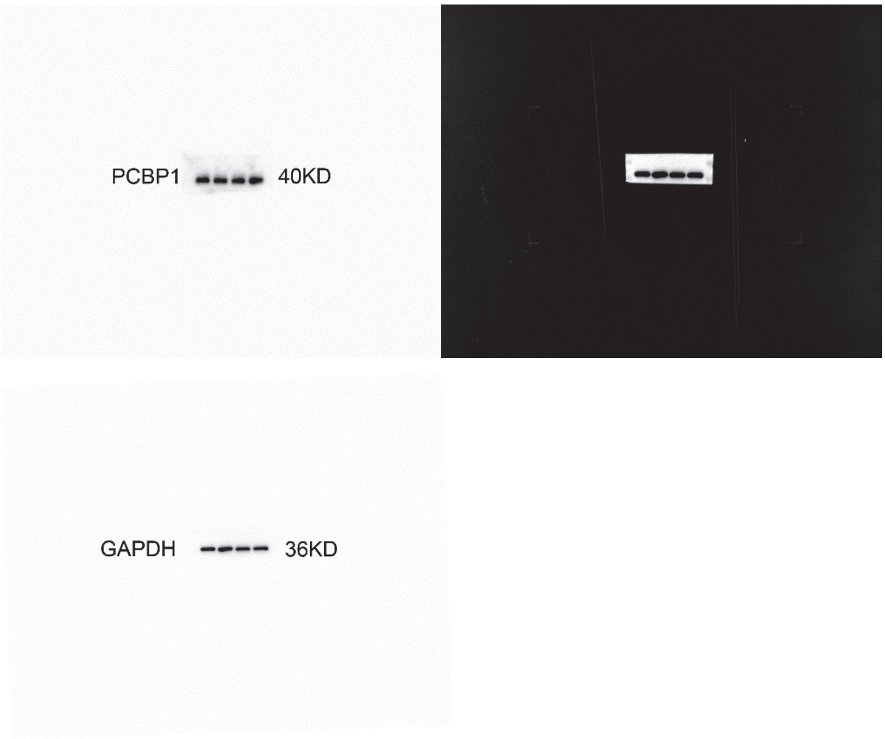

Figure6I

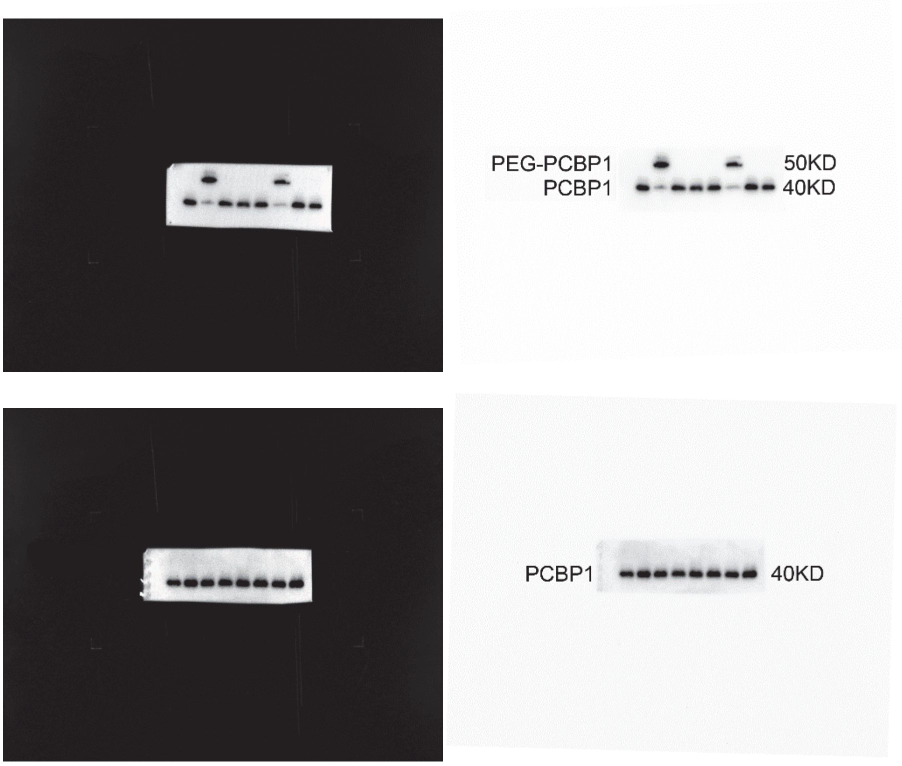

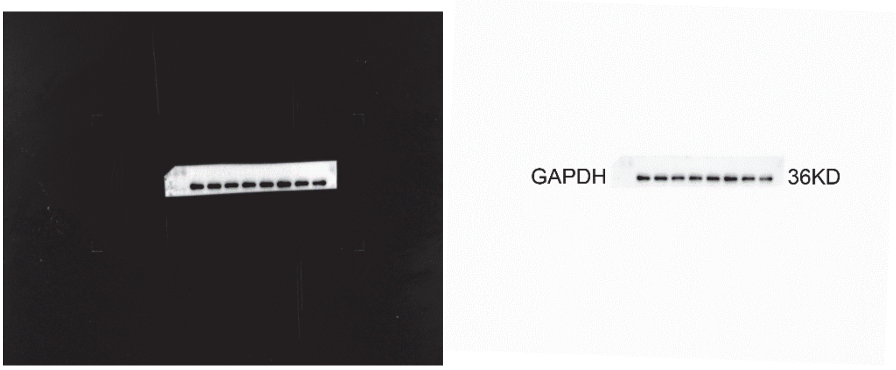

Figure6J

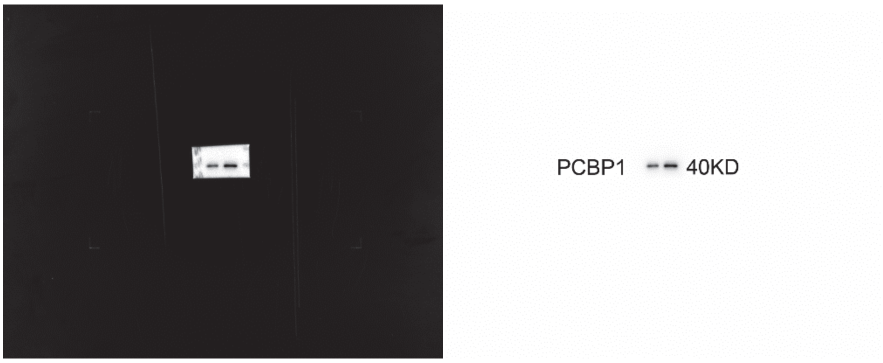

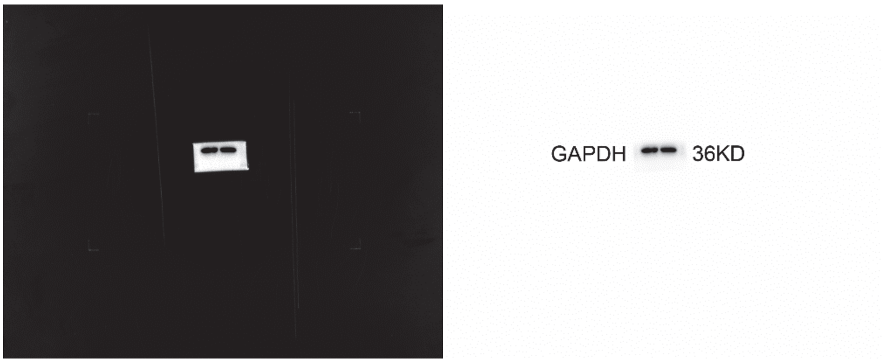

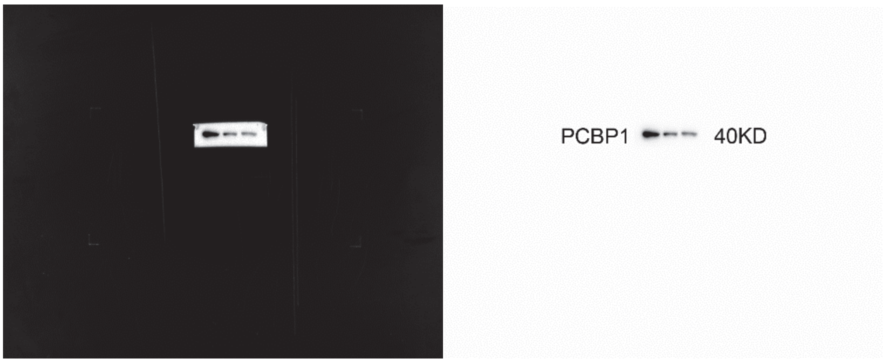

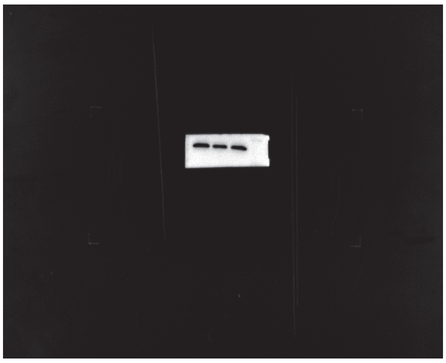

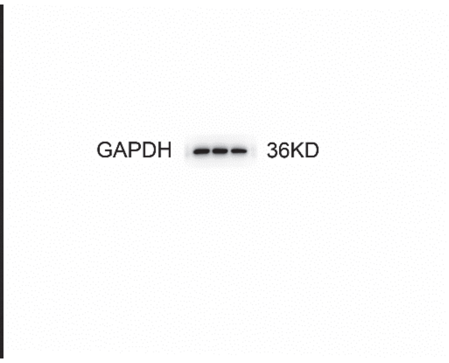

Figure6L

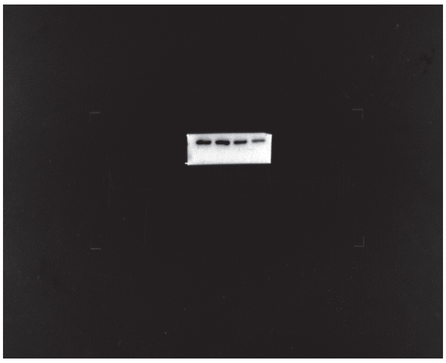

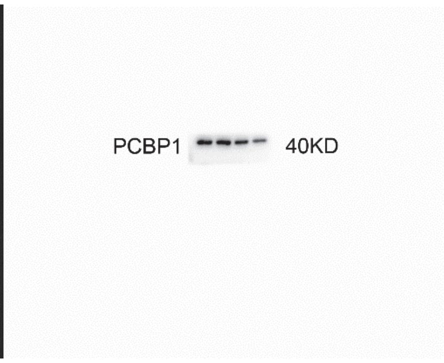

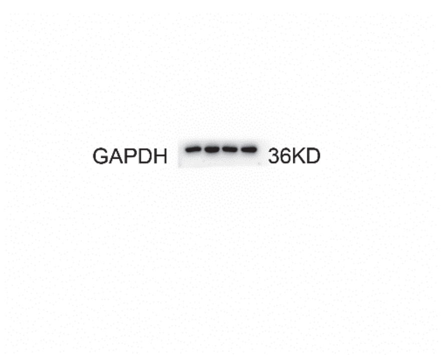

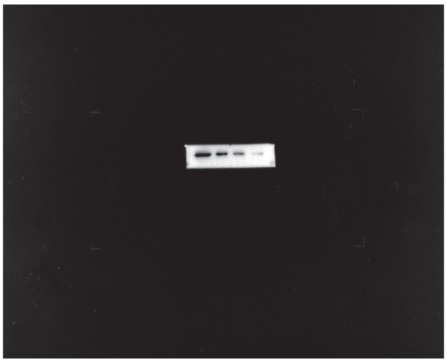

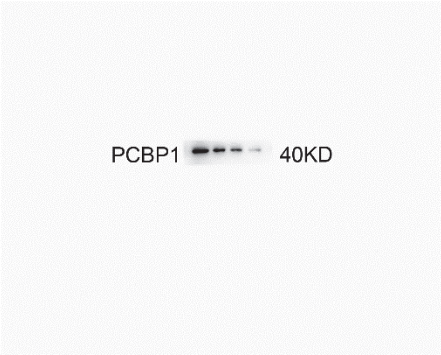

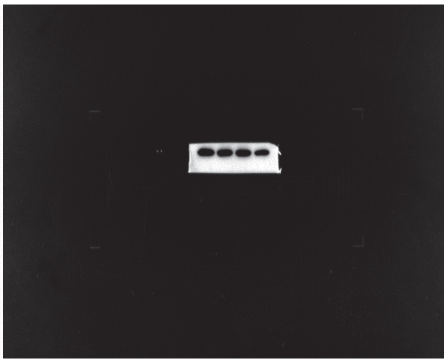

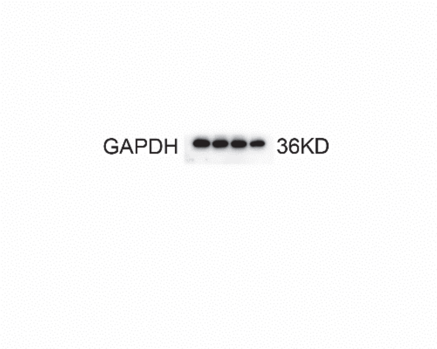

Figure6M

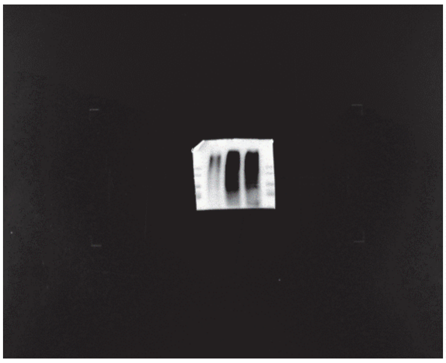

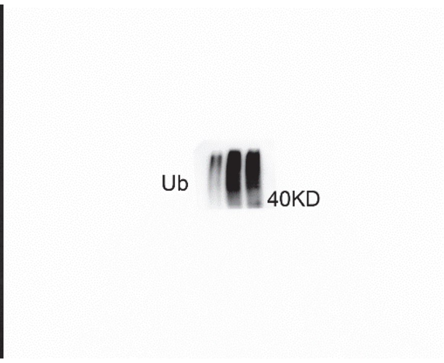

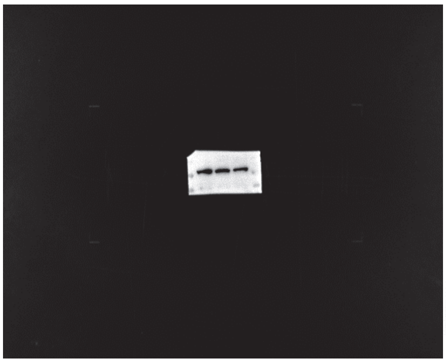

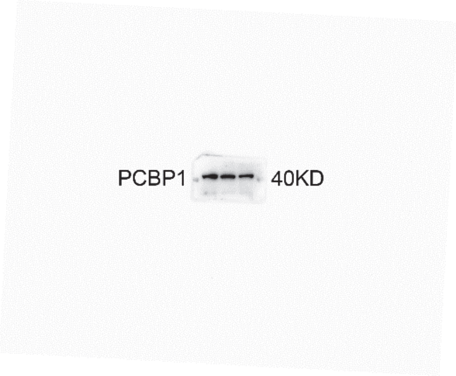

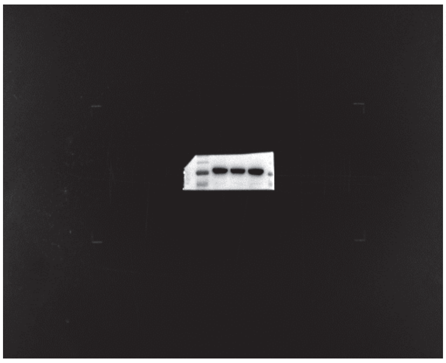

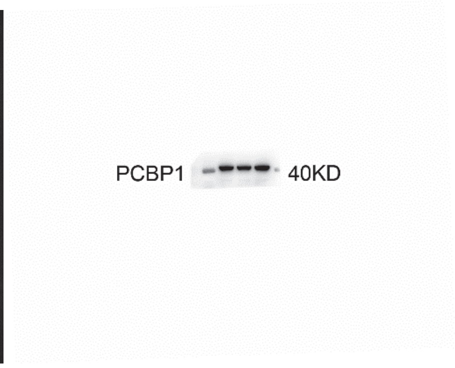

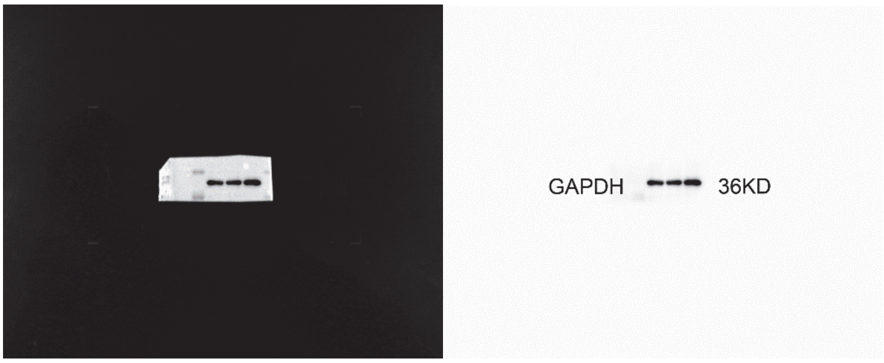

Figure7A

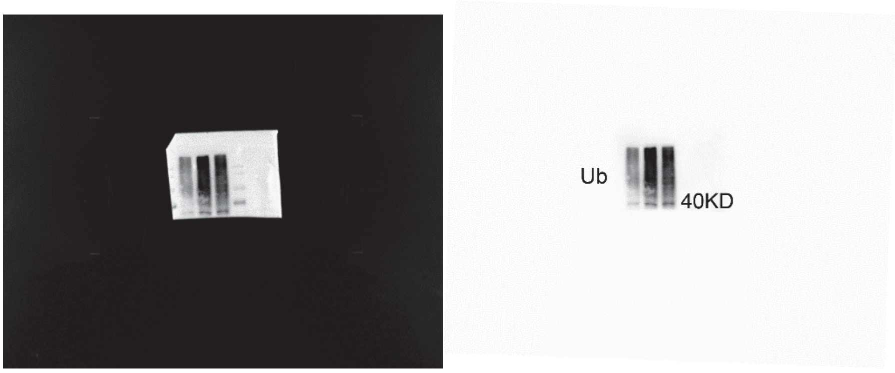

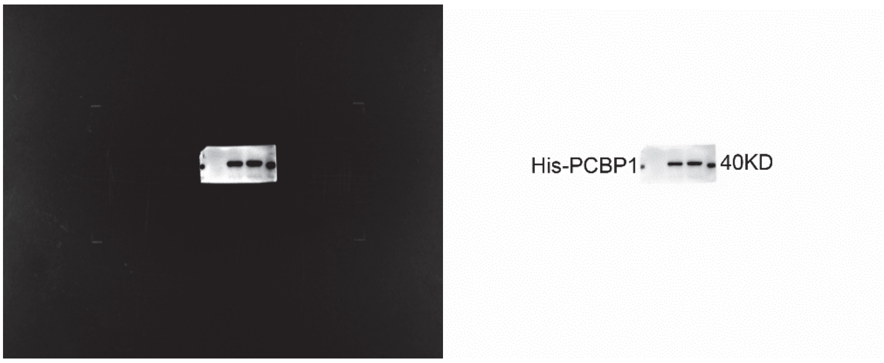

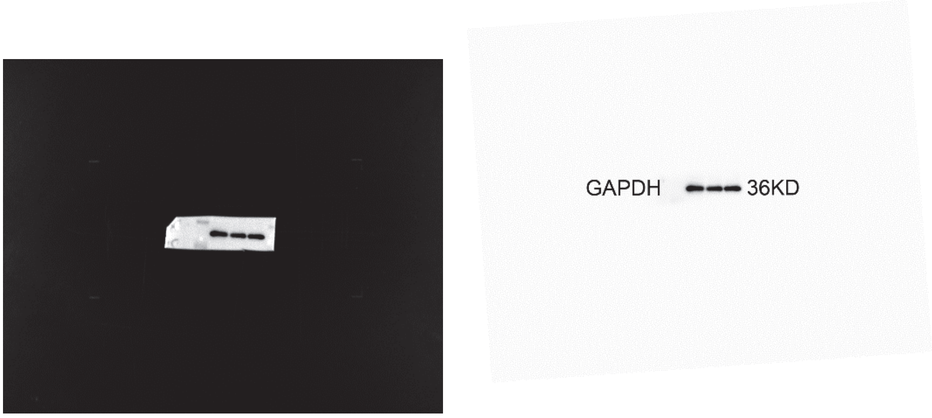

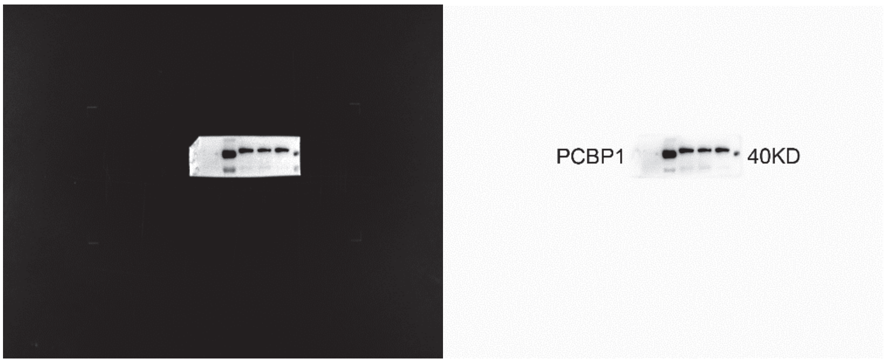

Figure7E

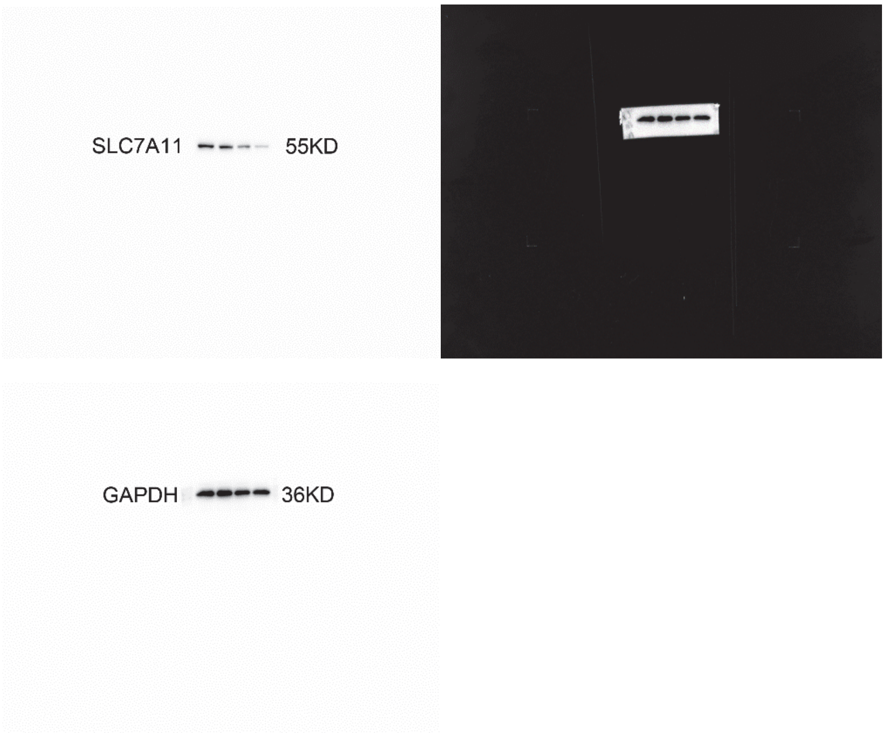

Figure7F

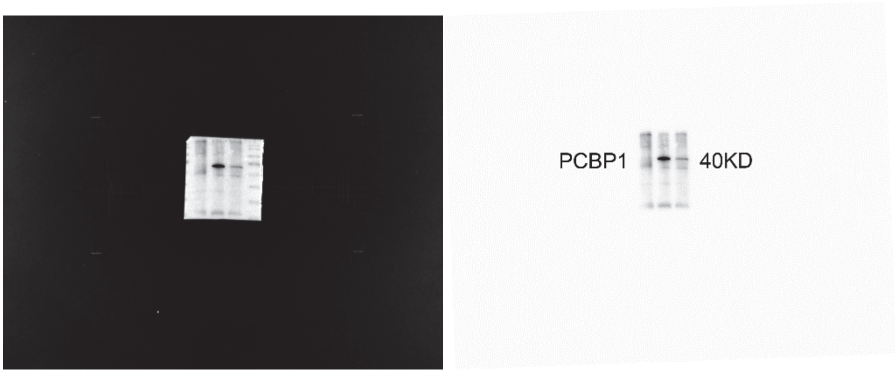

NOTE: Please save this file locally before filling in the table, DO NOT work on the file within your internet browser as changes will not be saved. Adobe Acrobat Reader (available free here) is recommended for completion.

ARRIVE

The ARRIVE guidelines 2.0: author checklist

The ARRIVE Essential 10

These items are the basic minimum to include in a manuscript. Without this information, readers and reviewers cannot assess the reliability of the findings.

Item		Recommendation	Section/line number, or reason for not reporting
**Study design**	1	For each experiment, provide brief details of study design including:	
		a. The groups being compared, including control groups. If no control group has been used, the rationale should be stated.	Control group: Subcutaneous tumor formation groups, tail vein injection-induced lung metastasis groups, spleen injection-induced liver metastasis groups, and intraperitoneal injection groups without protein overexpression or protein [illegible]
		b. The experimental unit (e.g. a single animal, litter, or cage of animals).	Single BALB/c nude mice (each mouse was independently implanted with tumor cells and received treatment, with no sharing of experimental conditions between
**Sample size**	2	a. Specify the exact number of experimental units allocated to each group, and the total number in each experiment. Also indicate the total number of animals used.	6 mice/group; total in experiment: 54 mice; total animals used: 54mice (no extra animals excluded before randomization).
		b. Explain how the sample size was decided. Provide details of any *a priori* sample size calculation, if done.	The sample size calculation method was determined based on the number of units used in actual experimental results.
**Inclusion and exclusion criteria**	3	a. Describe any criteria used for including and excluding animals (or experimental units) during the experiment, and data points during the analysis. Specify if these criteria were established *a priori*. If no criteria were set, state this explicitly.	Inclusion criteria: 6–8 weeks old BALB/c nude mice, weight 18–22 g, SPF-grade, no visible health abnormalities (e.g.skin lesions, abnormal activity); criteria established a priori before experiment initiation.
		b. For each experimental group, report any animals, experimental units or data points not included in the analysis and explain why. If there were no exclusions, state so.	Exclusion criteria: Mice with tumor implantation failure (tumor volume <50 mm³ at 7 days post-implantation), or accidental death during treatment (e.g., anesthesia [illegible]
		c. For each analysis, report the exact value of *n* in each experimental group.	n for analysis: 4-6 mice/group (2 tumor implantation failure, 2 accidental death）
**Randomisation**	4	a. State whether randomisation was used to allocate experimental units to control and treatment groups. If done, provide the method used to generate the randomisation sequence.	Randomisation used: Stratified randomization by body weight (mice stratified into 3 subgroups: 18–19 g, 19–20 g, 20–22 g); random sequence generated via Excel "Random Number Generation" function by an independent technician.
		b. Describe the strategy used to minimise potential confounders such as the order of treatments and measurements, or animal/cage location. If confounders were not controlled, state this explicitly.	Confounder control: Cage location randomized in the SPF facility (mice from different groups placed alternately on racks); treatment order randomized (mice from different groups processed in random order each day) to avoid batch effects.
**Blinding**	5	Describe who was aware of the group allocation at the different stages of the experiment (during the allocation, the conduct of the experiment, the outcome assessment, and the data analysis).	Only the independent technician was aware of group allocation (random sequence sealed in opaque envelopes).
**Outcome measures**	6	a. Clearly define all outcome measures assessed (e.g. cell death, molecular markers, or behavioural changes).	(1) Tumor volume (measured by vernier caliper, calculated as V = 0.5 × length × width²);<br>(2)The primary measurement in the [illegible]
		b. For hypothesis-testing studies, specify the primary outcome measure, i.e. the outcome measure that was used to determine the sample size.	Tumor volume (sample size calculated based on the expected difference in tumor volume between ASF1B or ZDHHC9 overexpression or protein knockdown group and Control group).
**Statistical methods**	7	a. Provide details of the statistical methods used for each analysis, including software used.	(1) Tumor volume: One-way analysis of variance (ANOVA) followed by Tukey's post-hoc test for multiple comparisons; (2) Data analyzed via SPSS 22.0 software.
		b. Describe any methods used to assess whether the data met the assumptions of the statistical approach, and what was done if the assumptions were not met.	Normality tested via Shapiro-Wilk test, variance homogeneity tested via Levene's test; if normality violated, Kruskal-Wallis H test (non-parametric) used instead.
**Experimental animals**	8	a. Provide species-appropriate details of the animals used, including species, strain and substrain, sex, age or developmental stage, and, if relevant, weight.	Species: Mus musculus (mouse); Strain: BALB/c nude mice; Sex: Half male, half female; Age: 6–8 weeks; Weight: 18–22 g.
		b. Provide further relevant information on the provenance of animals, health/immune status, genetic modification status, genotype, and any previous procedures.	Jiangsu Jicui Laboratory Animal Biology Co., Ltd.
**Experimental procedures**	9	For each experimental group, including controls, describe the procedures in enough detail to allow others to replicate them, including:	
		a. What was done, how it was done and what was used.	Tumor xenograft experiments:1×106 transfected cells were subcutaneously injected into the lower left forelimb. GC liver metastasis:A 5 × 106/20 μL cell suspension
		b. When and how often.	Tumor xenograft experiments:1×106 transfected cells were subcutaneously injected into the lower left forelimb. GC liver metastasis:A 5 × 106/20 μL cell suspension
		c. Where (including detail of any acclimatisation periods).	The operation location is the Animal Experiment Center of Anhui Medical University. The facility of this center is of Specific Pathogen-Free (SPF) grade, and
		d. Why (provide rationale for procedures).	The rationale for the operations is to clarify the biological effects of different models in the experiment:<br>The subcutaneous tumor formation
**Results**	10	For each experiment conducted, including independent replications, report:	
		a. Summary/descriptive statistics for each experimental group, with a measure of variability where applicable (e.g. mean and SD, or median and range).	Subcutaneous xenograft confirmed that overexpression of ASF1B significantly increased the volume and weight of subcutaneous tumors compared to the control group.Dissection of the liver [illegible]
		b. If applicable, the effect size with a confidence interval.	Tumor xenograft experiments:0.21 ±0.15g in control group VS 0.42±0.12g in ASF1B overexpression group.

安徽医科大学
LLSC20231296

编号：

安徽医科大学实验动物伦理委员会
课题论证报告
（正　本）

课题负责人：王明亮

课题名称：TLK2 通过抑制 ATF4 泛素化降解上调氨基酸代谢促进胃癌进展的机制研究

承担单位：安徽医科大学

一、伦理委员会对该课题方案进行了论证，并特别对以下三方面进行了认真讨论：

1、申请人资格和所用动物的品种品系、质量等级、规格是否合适，能否通过改良设计方案或用高质量的动物来减少所用动物的数量；

2、该项目是否必须用实验动物进行实验，即能否用计算机模拟、细胞培养等非生命方法替代动物或用低等动物替代高等动物进行实验；

3、能否通过改进实验方法、调整实验观测指标、改良处死动物的方法，来优化实验方案、善待动物。

二、同意实施该课题方案。实施过程中严格按照已申请实验方案实施。

三、课题方案如需修改，须事先经伦理委员会论证方可实施，修改内容及其原因需详细备案。

四、实施过程中如出现任何不良反应需立即向伦理委员会做出书面报告。

生效日期：2023 年 4 月 20 日

同意项目申报，获资助后另行伦理审查

安徽医科大学实验动物伦理委员会

（公章）

安徽医科大学实验动物伦理委员会

编 号： 2023451

安徽医科大学第一附属医院临床医学研究伦理委员会
课题论证报告
（正　本）

课题负责人：王明亮

课题名称：TLK2 通过抑制 ATF4 泛素化降解上调氨基酸代谢促进胃癌进展的机制研究

承担单位：安徽医科大学第一附属医院

一、伦理委员会对该课题方案进行了论证，并特别对以下三方面进行了认真讨论：

1、研究对象的权利与利益；

2、确保取得知情同意的措施；

3、存在的危险与可能的受益。

二、同意该课题的实施方案并申报 2023 年度安徽省教育厅高等学校科研计划项目。实施过程中请使用经论证的知情同意书、问卷、说明信等材料。

三、课题方案如需修改，须事先经伦理委员会论证方可实施，修改内容及其原因需详细备案。

四、实施过程中如出现任何不良反应需立即向伦理委员会做出书面报告。

生效日期：2023 年 4 月 20 日

安徽医科大学第一附属医院临床医学研究伦理委员会

（盖 章）

安徽医科大学第一附属医院 临床医学研究伦理委员会
